# Collaborative Care for Chronic Pain After Traumatic Brain Injury: A Randomized Clinical Trial

**DOI:** 10.1001/jamanetworkopen.2024.13459

**Published:** 2024-06-03

**Authors:** Jeanne M. Hoffman, Mary Curran, Jason Barber, Sylvia Lucas, Jesse R. Fann, Jennifer M. Zumsteg

**Affiliations:** 1 Department of Rehabilitation Medicine, University of Washington School of Medicine, Seattle; 2 Department of Neurological Surgery, University of Washington School of Medicine, Seattle; 3 Department of Neurology, University of Washington School of Medicine, Seattle; 4 Department of Psychiatry and Behavioral Sciences, University of Washington School of Medicine, Seattle; 5 Valley Medical Center, Seattle, Washington

## Abstract

**Importance:**

Chronic pain after traumatic brain injury (TBI) is prevalent and associated with poor outcomes. By providing multidisciplinary care through expert consultation, a collaborative care (CC) treatment approach may reduce pain interference.

**Objective:**

To compare CC with usual care (UC) in decreasing pain interference.

**Design, Setting, and Participants:**

This randomized clinical trial was conducted from July 2018 through April 2021 at 2 hospital-based academic rehabilitation medicine clinics in Seattle, Washington. Participants included adults with mild-to-severe TBI (at least 6 months before enrollment) and chronic pain. Data analysis was performed from March 30, 2022, to August 30, 2023.

**Intervention:**

The CC intervention (called TBI Care) included up to 12 in-person or telephone visits over 16 weeks with a care manager (CM) who provided person-centered cognitive behavioral treatment. The CM met weekly with members of the expert team to review participants and discuss recommendations to optimize treatment.

**Main Outcomes and Measures:**

The primary outcome was pain interference on the Brief Pain Inventory at treatment conclusion (4 months after randomization). Secondary outcomes included pain interference at 8 months; pain severity; symptoms of depression, anxiety, and sleep disturbance; pain-related emergency department visits; community participation; and participant satisfaction. Linear mixed-effects regression was used for analysis.

**Results:**

A total of 1379 individuals were screened for eligibility, and 158 were randomized (79 to CC and 79 to UC). The participants were mostly women (92 participants [58%]) with a mean (SD) age of 46.8 (13.2) years and a mean (SD) of 15.3 (3.0) years of education. TBI occurred a mean (SD) of 4.0 (5.9) years (median [IQR], 1.9 [0.8-4.5] years) before enrollment. All TBI severities were included, and of 149 participants for whom TBI severity was known, the majority (97 participants [65%]) had mild TBI. In the CC group, 71 participants (90%) completed at least 11 sessions, and, at 4 months, this group had significantly lower pain interference scores compared with the UC group (mean [SD], 3.46 [2.17] vs 5.03 [2.28]). This difference was maintained at 8 months after randomization, with mean (SD) TBI care pain interference scores of 3.61 (2.22) for CC vs 4.68 (2.51) for UC. At 4 months, there was significantly lower pain severity in the CC group vs UC group (mean [SD] score, 3.63 [1.95] vs 4.90 [1.96]), as well as symptoms of depression (mean [SD] score, 8.07 [5.34] vs 11.31 [6.37]) and anxiety (mean [SD], 6.20 [5.17] vs 9.58 [6.00]). Satisfaction with pain treatment (mean [SD] score, 2.99 [1.23] vs 2.52 [1.25]), clinical care (mean [SD] score, 3.28 [1.00] vs 2.84 [1.26]), and overall health care (mean [SD] score, 3.25 [0.88] vs 2.82 [1.00]) were significantly higher in the CC group vs the UC group; global impression of change was significantly lower in the CC group vs the UC group (mean [SD] score, 2.74 [1.02] vs 3.47 [1.26]) (lower scores denote a better impression of change).

**Conclusions and Relevance:**

In this randomized clinical trial of CC compared with UC for patients with TBI, CC was effective at reducing pain interference and was sustained at 8-month follow-up. Further research is needed to examine the implementation and cost-effectiveness of CC for TBI in other health care settings.

**Trial Registration:**

ClinicalTrials.gov Identifier: NCT03523923

## Key Points

**Question:**

Can collaborative care, an integrated, team-driven approach to delivering patient-centered, evidence-based treatment, improve chronic pain interference for individuals with traumatic brain injury (TBI)?

**Findings:**

In this randomized clinical trial comparing collaborative care with usual care for 158 adults with TBI, patients randomized to collaborative care had greater improvement in pain interference, symptoms of depression and anxiety, and satisfaction with care. Some improvements were maintained up to 8 months after randomization.

**Meaning:**

Collaborative care is a promising intervention for the treatment of chronic pain, including headache, for individuals with TBI.

## Teaser

This randomized clinical trial compares a collaborative care intervention vs usual care in decreasing chronic pain interference for patients with traumatic brain injury.

## Introduction

Chronic pain, defined as pain experienced more than one-half of the days for at least 3 months, is present in more than one-half of individuals following traumatic brain injury (TBI) of any severity^1^ and includes headache,^2,3,4^ musculoskeletal, and neuropathic pain.^5,6^ In a study of moderate-to-severe TBI,^7^ approximately 46% of 3804 participants endorsed current chronic pain, with back pain occurring most frequently (65%) followed by head pain (47%). Participants reported a median of 4 pain locations, suggesting a complex picture of pain.^7^ The prevalence of pain symptoms after TBI and their association with poor outcomes highlight chronic pain management as an important treatment target after TBI.^8,9^ There are currently no clinical practice guidelines for chronic pain treatment after TBI. In our own previous work,^5^ pain was endorsed by 73% of individuals 1 year after moderate-to-severe TBI, with 55% reporting pain interference with important daily activities. Chronic pain has been found to disrupt cognition, activities of daily living, sleep, mood, and social engagement in individuals with TBI and other populations.^10,11,12,13,14,15,16,17^

Multiple barriers exist to access clinicians with TBI expertise, including lack of coordinated communication across clinicians; travel distance to clinicians; financial barriers; impact of cognitive, psychologic, and physical barriers to care; and social support limitations. Therefore, patients with chronic pain and TBI often do not receive effective, coordinated treatment.^18,19,20,21^

Given the multiple types of posttraumatic pain (eg, headache, musculoskeletal, and neuropathic)^22^ that individuals with TBI experience, as well as common comorbid conditions, such as depression, anxiety, and sleep difficulties, optimal treatment needs to be accessible, multimodal, and matched to current patient needs and goals. Ideally, this is a shared decision-making process based on validated symptoms measures. Therefore, with input from persons with TBI and the expertise of our TBI specialists, we opted to test a collaborative care (CC) approach to treatment of chronic pain, including headache, in individuals with TBI.

CC is a patient-centered, team-based approach to providing evidence-based care. CC includes coordination of services and proactive outreach to engage, activate, and promote self-management and treatment adherence toward specified treatment targets.^23^ Importantly, CC ensures timely treatment adjustments based on ongoing assessment of response to treatment. CC was developed to deliver coordinated physical and mental health care within a primary care setting and has been adapted for a wide range of clinical settings and conditions. Multiple high-quality clinical trials, meta-analyses, and systematic reviews^24,25,26,27,28^ have demonstrated that CC is an effective approach to treating depression, anxiety, PTSD, substance abuse, and other chronic conditions in diverse medical settings. Several rigorous clinical trials^29,30,31^ also suggest that it is beneficial in chronic pain.

To the best of our knowledge, this is the first study to use the CC approach to treat chronic pain in individuals with TBI. Given the lack of clinical practice guidelines for the treatment of pain after TBI, our team monitored and updated recommendations for medications targeting each participant’s pain conditions (eg, headache, neuropathy, or osteoarthritis) in the context of TBI and emphasized nonpharmacological pain management. Our version of CC, called TBI Care, used a care manager (CM; M.C.) to provide cognitive behavioral interventions for pain, modified as appropriate for individuals with cognitive difficulties commonly seen after TBI and individually tailored to specific needs. CC was set within rehabilitation medicine clinics to maximize the likelihood that TBI-trained clinicians would address comorbid conditions. We tested the hypothesis that CC vs usual care (UC) would reduce pain interference, as measured by the Pain Interference Scale of the Brief Pain Inventory (BPI),^32^ which assesses the extent to which pain hinders engagement in important life activities, sleep, and enjoyment of life. Secondary aims focused on the effectiveness of CC on decreasing pain intensity, comorbid depression and anxiety symptoms, sleep difficulties, utilization of emergency department (ED) visits, and increasing community participation and satisfaction with care.

## Methods

We conducted a randomized clinical trial comparing CC with UC in 2 hospital-based academic rehabilitation clinics at Harborview Medical Center and the University of Washington Medical Center–Montlake Campus (both in Seattle, Washington). This study was approved by the institutional review boards before enrollment of the first participant. Those who met inclusion and exclusion criteria provided written informed consent. This report follows the Consolidated Standards of Reporting Trials (CONSORT) reporting guidelines for randomized studies.

### Participant Eligibility

Clinic patients, aged 18 years or older, were eligible to be in the study if they had a definitive diagnosis of mild-to-severe TBI according to medical record review with a diagnosis provided by a physiatrist. They needed to report experiencing moderate or higher chronic pain (a score of 4 or higher on a numeric rating scale ranging from 0, no pain, to 10, worst pain), for at least 6 months. They also needed to have an appointment or have been seen in the last 12 months by a brain injury clinician, were willing to accept additional help with their pain, were able to read and speak English, had the ability to communicate by telephone, and provided informed consent. We excluded participants with substantial cognitive impairment, assessed as answering more than 1 incorrect response on the Six-Item Screener^33^; having a terminal illness or pain associated with a cancer diagnosis; having major surgery anticipated during the study period; or having a diagnosis of bipolar disorder with psychotic features or current psychotic disorder.

### Recruitment

Appointment lists were screened for inclusion criteria (TBI diagnosis and chronic pain), or participants were referred to the study by their clinic practitioner. Once potential participants were identified, they received a letter explaining the study and then were approached either during a clinic visit or via telephone to assess their interest.

### Enrollment and Randomization

Participants were enrolled from July 2018 through April 2021. The study began 18 months before COVID-19 emergency clinical measures were instituted and ended during the COVID-19 pandemic. Screening procedures were similar before and after clinical adaptations to the COVID-19 outbreak. Enrolled participants who consented and completed a baseline assessment were randomized to CC or UC (Figure). The trial protocol in shown in Supplement 1. Randomization was embedded in the screening database and was conducted in blocks of 4. Once the participant was randomized, the CM was notified of their group. Those randomized to CC worked with the CM for up to 12 sessions (in-person, telephone, or video sessions) over a 16-week treatment period and completed a 24-week postrandomization check-in call with the CM. Participants assigned to UC were informed of the assignment via telephone by an unblinded research team member (usually the CM) and were mailed a letter explaining the assignment and a list of resources related to brain injury, pain, and mental health. The CM contacted participants assigned to CC to arrange the timing of their first treatment session.

**Figure.**
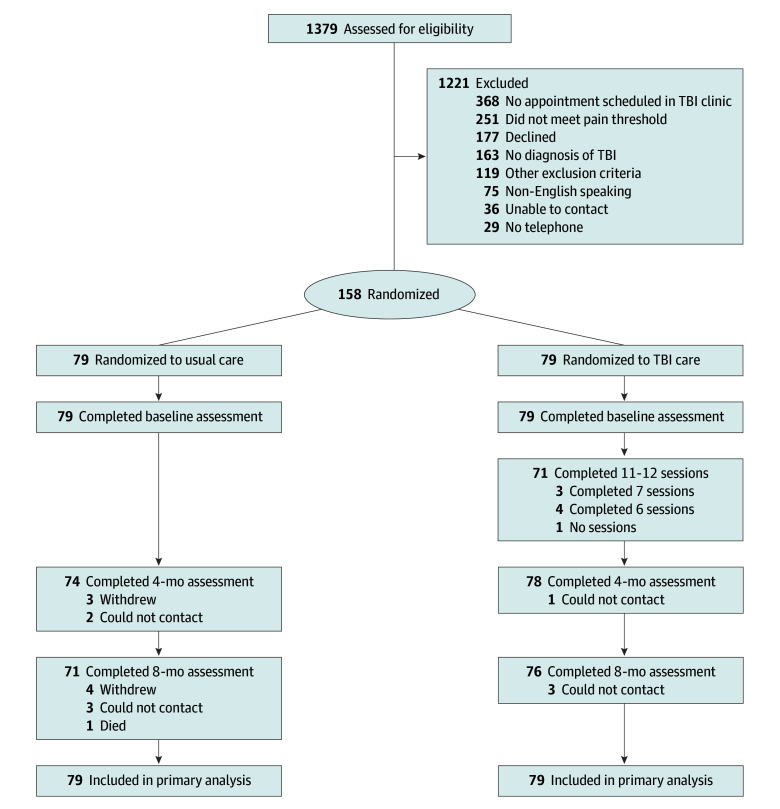
CONSORT Diagram TBI indicates traumatic brain injury.

### Intervention

The CC intervention is described in Table 1. Briefly, CC was organized around the participant, the CM, the participant’s rehabilitation clinic practitioner, and the study’s expert team. The CM (a trained masters-level social worker) provided cognitive behavioral treatment, care management, and support for collaborative medical management. Pain severity and interference were assessed at every session, and depression, anxiety, and sleep were assessed at the first, fourth, seventh, and tenth sessions using the Patient Health Questionnaire–9 (PHQ-9)^34^ and Generalized Anxiety Disorder Questionnaire–7 (GAD-7)^35^ and were documented in an Excel spreadsheet (version 2019; Microsoft). The CM met for an hour weekly with members of a team of TBI subject matter experts (psychologist, J.M.H.; physiatrist, J.M.Z.; psychiatrist, J.R.F.; and headache specialist, S.L.) to review active participants’ progress and discuss treatment recommendations.

**Table 1.** Overview of TBI Care Collaborative Care Intervention

Characteristic	Description
Model	
Collaborative care approach using evidence-based CBT strategies for pain	Manualized guide with outline of weekly session elements and stand-alone modules, tailored to participant goals and needs Participant workbook (paper and/or electronic) and relaxation recordings provided for practice between sessions Up to 12 sessions over 16 wk, 30-60 min each; choice of in person, telephone, video, or combination (in-person sessions stopped during COVID-19)
Structure	
Weekly session structure	Assess treatment response weekly via pain measures and every 4 wk with depression, anxiety, and sleep measures Review participant’s adherence to nonpharmacological and pharmacological treatments Address barriers to adherence, using problem-solving and motivational enhancement strategies Discuss any new treatment recommendations from the TBI Care team (eg, CBT strategies, possible referrals, initiation of new medication or dose adjustments, and so forth) Review home activities, including their practice and application of pain self-management skills Instruction and rehearsal of CBT self-management skills; encourage practice between sessions
Initial session module	Overview of TBI Care and pain self-management: education, assessment, treatment goals, and introduction of relaxation skills
Second session module	Goal setting and staying active
Middle session modules (sessions 3-11)	Flexible modular approach: tailored and paced, based on goals, including problem-solving skills, physical activity and pain, pacing and energy management, relaxation skills, coping with sleep issues, social and community support, working with thoughts around pain, mindfulness strategies, managing emotions, and coping with pain flares
Last session	Maintaining skills after treatment and developing ongoing pain self-management plan
8-wk Follow-up call	20-30 min Check-in regarding maintaining and integrating pain management skills
CM consultation and coordination	Weekly consultation, including members of the TBI expert team Recommendations for treatment adjustment based on pain interference, symptom severity, and response As indicated, CM provided the participant’s relevant health care clinician (eg, physiatrist, primary care) with treatment recommendations from the expert team and supported coordinated care As indicated, treatments included referral to other specialty clinics and also targeted comorbid conditions that likely contribute to pain interference or intensity

Abbreviations: CBT, cognitive behavioral treatment; CM, care manager; TBI, traumatic brain injury.

### Data Collection and Measurement Techniques

Participant demographics, TBI severity, and pain experience were collected at baseline assessments. Data on race and ethnicity were self-reported by participants and are included in this analysis because they describe the population included in the study. All outcome assessments were conducted by telephone at baseline, 4 months (primary), and 8 months (secondary) after randomization using structured interviews conducted by trained research study staff who were unaware of the participant’s group assignment. At each assessment period, medications were reviewed, including those currently prescribed (from the medical record) and any others reported being used by participants.

### Outcome Measures

The primary outcome was difference in pain interference at end of treatment (4 months) on the Pain Interference Scale from the BPI,^32^ which measures interference with general activity, mood, walking, work (outside or in home), relationships, sleep, and enjoyment of life. The score ranges from 0 (does not interfere) to 10 (interferes completely).

Secondary outcomes included pain interference at 8 months, in addition to the other outcomes measured at both 4 and 8 months. Pain intensity was measured by the Brief Pain Intensity–4 subscale of the BPI,^32^ which asks about pain over the past 7 days on current, worst, average, and least on a scale of 0 (no pain) to 10 (worst pain). The Brief Pain Intensity–4 was collected 4 times over the course of 1 week, and all items and all time points were averaged to capture pain intensity. Symptoms of depression were assessed using the PHQ-9,^34^ anxiety was assessed using the GAD-7,^35^ and sleep disturbance was assessed using the Pittsburgh Sleep Quality Index.^36^ Frequency of pain-related ED visits that occurred across the region were collected from the Emergency Department Information Exchange for the 4 months before randomization and the 4 months before each assessment period. We also assessed community participation using the Participation Assessment with Recommended Tools-O-17.^37^ Exploratory outcomes included assessment of satisfaction with brain injury rehabilitation clinic care with the Patient Global Assessment of Treatment Satisfaction,^38^ with higher scores reflecting higher satisfaction and a patient self-assessment of improvement in pain from the start of the study using the Patient Global Impression of Change,^39^ which is a single item ranging from 1 (very much better) to 7 (very much worse).

### Sample Size Calculation

We based our sample size estimate on 2 prior collaborative care studies^40,41^ in neurorehabilitation populations in which pain interference was a secondary outcome. On the basis of effect sizes from those studies of 0.55 and 0.51 (a medium effect size *d* in Cohen^42^ terminology) and using a 2-sample *t* test, a sample size of 63 per group would provide 80% power to detect about the same difference (Cohen *d* = 0.50) between CC and UC in reduction of pain interference from baseline to 4 months. To be conservative, we recruited 158 participants, planning for a yield of at least 126 or 63 per group for the analytic sample, assuming an 80% retention rate.

### Statistical Analysis

Data analysis was performed from March 30, 2022, to August 30, 2023. Differences in patient baseline characteristics between the 2 treatment groups were quantified using standardized mean differences. The primary outcome, pain interference on the BPI, was analyzed using the intent-to-treat principle. All outcomes were modeled longitudinally using mixed-effects linear regression, using all assessments at baseline, 4 months, and 8 months (see statistical analysis plan in the trial protocol in Supplement 1). Poisson regression was used to model the number of ED visits. Participant effects were modeled using random intercepts, and time was modeled as a categorical effect. Effect sizes were expressed as the group difference in the change from baseline and were modeled using a group-by-time interaction. The Kenward-Roger approximation was used to estimate the *F *test degrees of freedom. All models adjusted for baseline PHQ-9 and GAD-7 scores, and additional models also included a main effect for whether the assessment occurred before vs after the start of the COVID-19 pandemic.

A 2-sided threshold of *P* < .05 was used to define statistical significance, and no adjustments were made for multiple comparisons. All analyses were performed using SPSS statistical software version 26 (IBM) and SAS statistical software version 9.4 (SAS Institute).

## Results

### Participants

In total, 1379 individuals were screened for eligibility, resulting in 158 randomized, with 79 in each group (Figure). The sample included 92 women (58%), with a mean (SD) age of 46.8 (13.2) years, a mean (SD) of 15.3 (3.0) years of education, and a mean (SD) of 4.0 (5.9) years (median [IQR], 1.9 [0.8-4.5] years) postinjury. Patients primarily had mild TBI (97 of 149 participants [65%] for whom TBI severity was known). With regard to race, 1 participant (1%) was American Indian or Alaska Native, 9 participants (6%) were Asian, 9 participants (6%) were Black or African American, 1 participant (1%) was Native Hawaiian or Pacific Islander, 124 participants (79%) were White, 6 participants (4%) were more than 1 race, 7 participants (4%) were other races (self-defined by participants), and 1 participant (1%) was of unknown race; with regard to ethnicity, 8 participants (5%) were Hispanic, 147 participants (95%) were non-Hispanic, and 3 (2%) were of unknown ethnicity. No significant differences were found between groups in terms of demographic or clinical characteristics (Table 2). There were no significant differences between groups at baseline regarding pain interference, pain intensity, number of pain locations, symptoms of depression or anxiety, sleep difficulties, or ED visits. The UC group reported significantly more back pain than the CC group (68 participants [86%] vs 56 participants [71%]), but both groups reported having more than 7 pain sites on average.

**Table 2.** Patient Characteristics

Characteristic	Patients, No. (%)^a^	
	Collaborative care (n = 79)	Usual care (n = 79)
Age, y		
Mean (SD)	47.1 (13.2)	46.4 (13.3)
Median (IQR) [range]	47.2 (36.7-57.0) [20.5-76.5]	46.2 (36.1-57.2) [21.6-74.3]
Time since injury, y		
Mean (SD)	3.2 (4.2)	4.8 (7.3)
Median (IQR) [range]	1.6 (0.7-2.8) [0.5-18.2]	2.1 (0.8-5.2) [0.2-43.1]
Unknown	0	1
Sex		
Male	34 (43)	32 (41)
Female	45 (57)	47 (59)
Race		
American Indian or Alaska Native	0	1 (1)
Asian	7 (9)	2 (3)
Black or African American	5 (6)	4 (5)
Native Hawaiian or Pacific Islander	0	1 (1)
White	60 (77)	64 (81)
Multiple races	2 (3)	4 (5)
Other^b^	4 (5)	3 (4)
Unknown	1	0
Hispanic ethnicity		
Hispanic or Latino	5 (6)	3 (4)
Not Hispanic or Latino	72 (94)	75 (96)
Unknown	2	1
Marital status		
Never married or single	15 (19)	24 (30)
Married	35 (45)	22 (28)
Domestic partnership	6 (8)	9 (11)
Separated	2 (3)	4 (5)
Divorced	16 (21)	17 (22)
Widowed	4 (5)	3 (4)
Unknown	1	0
Education duration, y		
Mean (SD)	15.4 (3.0)	15.2 (3.1)
Median (IQR) [range]	16 (14-17) [6-24]	15 (13-17) [6-24]
Unknown	5	5
Employment status		
Working now	22 (28)	28 (36)
Temporarily laid off	2 (3)	0
Sick leave or parental leave	2 (3)	1 (1)
Unemployed, looking for work	13 (17)	12 (15)
Retired	8 (10)	6 (8)
Unemployed, disabled	19 (24)	27 (35)
Keeping house	6 (8)	0
Student	2 (3)	1 (1)
Other	4 (5)	3 (4)
Unknown	1	1
Injury severity		
Mild	50 (66)	47 (64)
Complicated mild	7 (9)	2 (3)
Moderate	6 (8)	3 (4)
Moderate to severe	2 (3)	4 (5)
Severe	11 (14)	17 (23)
Unknown	3	6
Baseline BPI interference score		
Mean (SD)	5.4 (2.1)	5.8 (1.9)
Unknown	0	1
Baseline BPI intensity score, mean (SD)	4.9 (1.8)	5.4 (1.6)
Pain location		
Head	74 (94)	71 (90)
Neck	65 (82)	67 (85)
Back	56 (71)	68 (86)
Shoulder	59 (75)	63 (80)
Legs and/or feet	57 (72)	50 (63)
Arms and/or hands	48 (61)	49 (62)
Hips	40 (51)	45 (57)
Face or jaw	42 (53)	40 (51)
Other location	29 (37)	23 (29)
Buttocks	29 (37)	21 (27)
Chest	22 (28)	22 (28)
Pelvic area or groin	20 (25)	22 (28)
Widespread pain and/or fibromyalgia	24 (30)	19 (24)
Abdomen	19 (24)	21 (27)
Total pain locations, mean (SD) [range]	7.4 (3.0) [1-14]	7.3 (2.8) [2-13]
Baseline scores, mean (SD)		
Patient Health Questionnaire–9	11.4 (6.0)	13.1 (6.2)
Generalized Anxiety Disorder–7	8.6 (5.1)	10.4 (6.4)
Pittsburgh Sleep Quality Index	10.7 (4.6)	11.5 (4.4)
Emergency department visits for pain, mean (SD), No.^c^	0.11 (1.39)	0.13 (0.40)

Abbreviation: BPI, Brief Pain Inventory.

^a^ Unknown values were not included in calculations of percentages.

^b^ The 7 individuals who chose other were asked to indicate their race; 3 individuals opted not to provide details, 1 stated “mut,” 1 stated “Caucasian and indigenous SE American,” 1 stated “Mexican,” and 1 stated “Scandinavian/European.”

^c^ Refers to visits 4 months before randomization; 1 collaborative care participant who had 21 overall visits has been excluded.

### Primary Outcome

In the CC group, 71 participants (90%) completed at least 11 sessions. At 4 months, this group had statistically significantly lower pain interference compared with the UC group (mean [SD] score, 3.46 [2.17] vs 5.03 [2.28]; B, −1.26; 95% CI, −1.93 to −0.59; *P* < .001) (Table 3).

**Table 3.** Primary and Secondary Outcomes

Outcomes	Score, mean (SD)		Modeled effect size, B (95% CI)^a^	*P* value
	Collaborative care	Usual care		
Primary				
Participants assessed at 4 mo, No. (%)	77 (97)	73 (92)	NA	NA
Pain interference at 4 mo	3.46 (2.17)	5.03 (2.28)	−1.26 (−1.93 to −0.59)	<.001
Secondary				
Participants assessed at 8 mo, No. (%)	75 (95)	71 (90)	NA	NA
Pain interference at 8 mo	3.61 (2.22)	4.68 (2.51)	−0.71 (−1.38 to −0.03)	.04
Assessments at 4 mo				
Pain severity	3.63 (1.95)	4.90 (1.96)	−0.85 (−1.32 to −0.37)	.001
PHQ-9	8.07 (5.34)	11.31 (6.37)	−1.90 (−3.65 to −0.14)	.03
GAD-7	6.20 (5.17)	9.58 (6.00)	−1.79 (−3.38 to −0.19)	.03
PSQI	9.22 (4.57)	10.62 (4.61)	−0.78 (−1.90 to 0.34)	.17
ED visits related to pain in past 4 mo, mean (SD), No.^b^	0.05 (0.27)	0.09 (0.33)	0.63 (0.14 to 2.91)^c^	.56
PART-O-17	1.83 (0.61)	2.06 (0.61)	0.03 (−0.11 to 0.18)	.65
PGATS with treatment for pain	2.99 (1.23)	2.52 (1.25)	0.48 (0.10 to 0.85)	.01
PGATS with health care at University of Washington rehabilitation clinic	3.28 (1.00)	2.84 (1.26)	0.44 (0.09 to 0.80)	.01
PGATS overall satisfaction with all health care received	3.25 (0.88)	2.82 (1.00)	0.43 (0.11 to 0.76)	<.01
PGIC of pain now since started study	2.74 (1.02)	3.47 (1.26)	−0.74 (−1.13 to −0.34)	<.001
Assessments at 8 mo				
Pain severity	3.51 (2.07)	4.40 (2.27)	−0.45 (−0.93 to 0.03)	.07
PHQ-9	7.74 (4.86)	10.76 (6.40)	−1.74 (−3.51 to −0.02)	.03
GAD-7	6.59 (5.27)	8.52 (6.00)	−0.42 (−2.02 to 1.19)	.61
PSQI	8.48 (4.27)	9.69 (4.50)	−0.64 (−1.77 to 0.49)	.27
ED visits related to pain in past 4 mo^b^	0.11 (0.42)	0.20 (0.52)	0.63 (0.19 to 2.11)^c^	.45
PART-O-17	1.04 (0.61)	1.16 (0.72)	0.05 (−0.10 to 0.19)	.61
PGATS with treatment for pain	2.83 (1.12)	2.86 (1.08)	−0.02 (−0.40 to 0.35)	.90
PGATS with health care at University of Washington rehabilitation clinic	2.91 (1.00)	2.93 (1.05)	−0.01 (−0.37 to 0.35)	.94
PGATS overall satisfaction with all health care received	3.15 (0.89)	2.91 (1.11)	0.24 (−0.08 to 0.57)	.14
PGIC since study started	2.81 (1.12)	3.30 (1.41)	−0.49 (−0.89 to −0.09)	.02

Abbreviations: ED, emergency department; GAD-7, Generalized Anxiety Disorder–7; NA, not applicable; PART-O-17, Participation Assessment With Recommended Tools; PGATS, Patient Global Assessment of Treatment Satisfaction; PGIC, Patient Global Impression of Change; PHQ-9, Patient Health Questionnaire–9; PSQI, Pittsburgh Sleep Quality Index.

^a^ B refers to the fixed time-by-group interaction effect in a mixed-effects regression model fit using 3 time points (baseline, 4 months, and 8 months) that also included fixed effects for time (categorical), group, baseline PHQ-9 score, and baseline GAD-7 score, as well as a random participants intercept. Linear regression was used for continuous outcomes, logistic regression was used for dichotomous outcomes, and Poisson regression was used for counts (ED visits). No adjustments have been made for multiple comparisons

^b^ One collaborative care participant who had 21 overall visits has been excluded.

^c^ Data are risk ratio (95% CI).

### Secondary Outcomes

Analysis of secondary outcomes is presented in Table 3. The significant difference seen in pain interference at 4 months was maintained at 8 months (CC vs UC, mean [SD] score, 3.61 [2.22] vs 4.68 [2.51]; B, −0.71; 95% CI, −1.38 to −0.03; *P* = .04). The results showed that pain severity was significantly lower in the CC group vs the UC group at 4 months (mean [SD] score, 3.63 [1.95] vs 4.90 [1.96]; B, −0.85; 95% CI, −1.32 to −0.37; *P* = .001), along with having lower reports of depressive (mean [SD] score, 8.07 [5.34] vs 11.31 [6.37]; B, −1.90; 95% CI, −3.65 to −0.14; *P* = .03) and anxiety (mean [SD] score, 6.20 [5.17] vs 9.58 [6.00]; B, −1.90; 95% CI, −3.65 to −0.14; *P* = .03) symptoms. Individuals in the CC group also reported significantly higher satisfaction with treatment for pain (mean [SD] score, 2.99 [1.23] vs 2.52 [1.25]), clinical care (mean [SD] score, 3.28 [1.00] vs 2.84 [1.26]), and overall health care (mean [SD] score, 3.25 [0.88] vs 2.82 [1.00]), and lower global impression of change scores (mean [SD] score, 2.74 [1.02] vs 3.47 [1.26]) than those in the UC group at the end of the intervention.

At 8 months, significant differences were maintained between groups for reports of depressive symptoms (mean [SD] score, 7.74 [4.86] vs 10.76 [6.40]; B, −1.74; 95% CI, −3.51 to −0.02; *P* = .03) and for global impression of change in pain since the study started (mean [SD] score, 2.81 [1.12] vs 3.30 [1.41]; B, −0.49; 95% CI, −0.89 to −0.09; *P* = .02). We found no impact on treatment occurring before vs after the onset of the COVID-19 pandemic (results not shown).

Medications were categorized according to type and are shown in Table 4. There was minimal change in type of medications over time. Opioid use was reported for approximately 20% of participants (9 of 79 in the CC group and 22 of 79 in the UC group) at baseline and remained stable within and between groups across all assessment periods.

**Table 4.** Medication Summary

Medication type	Patients, No. (%) (N = 158)					
	Baseline		4-mo Assessment		8-mo Assessment	
	Collaborative care (n = 79)	Usual care (n = 79)	Collaborative care (n = 77)	Usual care (n = 73)	Collaborative care (n = 76)	Usual care (n = 71)
Other^a^	59 (75)	51 (65)	59 (77)	45 (62)	59 (78)	48 (68)
Antiepileptic	30 (38)	39 (49)	32 (42)	38 (52)	33 (43)	36 (51)
NSAID	30 (38)	31 (39)	35 (45)	24 (33)	32 (42)	24 (34)
Other (vitamin and/or mineral)	29 (37)	27 (34)	32 (42)	26 (36)	34 (45)	29 (41)
Cardiovascular	25 (32)	30 (38)	25 (32)	29 (40)	23 (30)	27 (38)
Sleep medication	23 (29)	30 (38)	23 (30)	27 (37)	23 (30)	25 (35)
Analgesic non-NSAID	19 (24)	20 (25)	21 (27)	20 (27)	26 (34)	19 (27)
Selective serotonin reuptake inhibitor	18 (23)	18 (23)	18 (23)	18 (25)	18 (24)	16 (23)
Muscle relaxant	13 (16)	22 (28)	17 (22)	20 (27)	17 (22)	21 (30)
Serotonin and norepinephrine reuptake inhibitor	16 (20)	19 (24)	17 (22)	18 (25)	19 (25)	20 (28)
Opioid	9 (11)	22 (28)	11 (14)	20 (27)	9 (12)	20 (28)
Acute headache	16 (20)	9 (11)	21 (27)	9 (12)	21 (28)	9 (13)
Antiemetic	11 (14)	13 (16)	13 (17)	14 (19)	11 (14)	14 (20)
Supplements for headache	10 (13)	14 (18)	12 (16)	11 (15)	13 (17)	12 (17)
Central nervous system stimulant	16 (20)	6 (8)	18 (23)	8 (11)	20 (26)	7 (10)
Tricyclic antidepressants	9 (11)	12 (15)	8 (10)	13 (18)	9 (12)	13 (18)
Benzodiazepine	9 (11)	9 (11)	6 (8)	8 (11)	9 (12)	8 (11)
Antihistamine	8 (10)	6 (8)	8 (10)	9 (12)	7 (9)	10 (14)
Antidepressant—other	5 (6)	8 (10)	5 (6)	9 (12)	5 (7)	9 (13)
Antipsychotic and/or mood stabilizer	4 (5)	7 (9)	5 (6)	9 (12)	5 (7)	9 (13)
Topical analgesic	4 (5)	6 (8)	7 (9)	7 (10)	6 (8)	8 (11)
Memory and/or cognition enhancing	7 (9)	2 (3)	8 (10)	5 (7)	8 (11)	6 (8)
Anxiolytic	0	4 (5)	1 (1)	5 (7)	1 (1)	5 (7)
Opioid antagonist	1 (1)	1 (1)	1 (1)	1 (1)	0	2 (3)
Calcitonin gene–related peptide monoclonal antibodies	0	1 (1)	2 (3)	4 (5)	3 (4)	6 (8)

Abbreviation: NSAID, nonsteroidal anti-inflammatory drug.

^a^ Other includes any medication not otherwise categorized, such as allergy or asthma medications and proton pump inhibitors.

## Discussion

Results of the current randomized clinical trial suggest that our CC intervention, TBI Care, had a significant positive impact on pain interference and pain severity compared with UC, with CC participants maintaining significantly lower pain interference scores up to 8 months after randomization. Pain interference scores were reduced by at least 30% in 56% of the CC group at 4 months and by 61% at 8 months compared with 27% and 38% at 4 and 8 months, respectively, in the UC group, suggesting sustained improvement. CC is a promising approach for the treatment of pain after TBI, especially given the high engagement and participant satisfaction in this study, along with the high level of pain complexity in this population, with multiple pain sites and comorbid conditions. Given that clinical practice guidelines are not currently available to guide pharmacologic or nonpharmacologic treatment of chronic pain after TBI, CC was designed to focus on patient-centered nonpharmacological approaches, including targeted cognitive behavioral treatment strategies, engagement in physical activity, and other behavioral interventions, with a goal to develop individual skills for symptom management in the reduction of pain interference. Similar to other CC trials, medication use was considered one of the many tools for pain self-management and was reviewed consistently by the expert team to make recommendations to participant’s clinicians. We adapted CC to address chronic pain after TBI by placing the primary focus on an individual’s use of evidence-based nonpharmacological approaches, which has been shown to decrease pain interference in daily life. In addition, we sought to encourage and engage patients toward meaningful goals in their lives.

The positive result for CC adds to the list of CC studies that have had a positive impact on pain in other rehabilitation populations^41^ and in other medical settings, such as primary care and oncology.^29,31,43,44^ There is a growing need for innovative care models to maximize population health outcomes given increasing health system complexity, worsening health care access, and negative outcomes following the COVID-19 pandemic. CC added substantial value over UC to both patients and rehabilitation care clinicians with experience in TBI.

Although the primary focus of CC was chronic pain treatment, the intervention group also received monitoring and treatment recommendations for common TBI-related comorbidities of depression, anxiety, and sleep disturbance and resulted in significant improvements in depressive and anxiety symptoms after treatment, with maintenance of lower reports of depressive symptoms up to 8 months after randomization. Although several secondary outcomes did not result in differences between groups, in particular ED visits and community participation, this may have been because of the timing of these assessments relative to change in pain interference. It is possible that the assessment period may not have been long enough to result in behavioral changes that might have led to changes in community participation and/or health care utilization. Ultimately, pain-related ED visits were generally low in both groups.

Importantly, CC is a promising intervention given changes in health care that resulted from COVID-19, with more treatment options available via telemedicine. During pandemic-altered operations, the use of technology generally increased in the study population, and the expert team reviewed participants using telephone or video-teleconferencing. CC offers a model to support a broad range of clinicians beyond those in rehabilitation medicine; our CM was able to facilitate care by connecting with primary care and mental health clinicians. Participants in this study had a wide range of TBI severity with multiple comorbidities, suggesting that CC can be effective across a diverse population of individuals with TBI.

### Limitations

Our study has several limitations. Participants were recruited from a brain injury rehabilitation clinic, and the results may not generalize to individuals with TBI without such specialty care. The majority of participants had mild TBI, were female, and completed some postsecondary education, which may differ from other clinic TBI populations. Although mild TBI is the most common severity of injury, TBI is more frequent in male individuals; therefore, further research powered and designed to detect differences in response related to severity of injury, sex, social determinants of health, and other factors is needed to consider further implementation and to build on the current study findings.

## Conclusions

These findings suggest that our CC intervention, TBI Care, is a promising approach to the treatment of chronic pain in individuals with TBI. The ability to engage in multimodal care with both skill development for self-management of chronic pain and symptom-guided expert adjustments based on response to treatment was associated with reduced pain interference that was maintained for many months after treatment was completed. Further research is needed to test the cost-effectiveness and implementation of TBI Care in a range of health care settings and to evaluate additional TBI-related comorbidities and outcomes.

## Notes

Supplement 1.
Trial Protocol and Statistical Analysis Plan

Supplement 2.
Data Sharing Statement
