# Supplementary material for: Collaborative Care for Chronic Pain After Traumatic Brain Injury: A Randomized Clinical Trial
Source: JAMA Netw Open. 2024 Jun 3;7(6):e2413459. doi: 10.1001/jamanetworkopen.2024.13459 (PMC11148690; doi:10.1001/jamanetworkopen.2024.13459)
Supplement: Supplement 1. — Trial Protocol and Statistical Analysis Plan [file jamanetwopen-e2413459-s001.pdf]

TBI Care Study
Manual of Procedures (MOP)

Contract Title: ***The Effectiveness of Collaborative Care versus Usual Care for Pain after Traumatic Brain Injury***
Informal Title: *TBI Care*

A 1:1 randomized controlled trial design will compare Collaborative Care (CC) to usual care (UC) to reduce pain interference among outpatients in our UW Medicine TBI clinics who have headache or chronic pain and meet other eligibility criteria.

Principal Investigator:
Jeanne M. Hoffman, Ph.D., University of Washington, Department of Rehabilitation Medicine

Supported by:
National Institute on Disability, Independent Living, and Rehabilitation Research (NIDILRR), University of Washington TBI Model System, Grant 90DPTB0008

IRB Committee B
#STUDY00003847

Contents

TBI Care Study Flow Chart....5
Questions Asked / Answered 6
IRB Submission and Approval Tracking Log....10
STUDY DESCRIPTION....14
TREATMENT CONDITIONS....14
- Usual Care group....14
- Collaborative Care Group....15
- Collaborative Care - Overview....15
- Data Analysis Methods....15

HIPAA General Guidelines and Confidentiality....15
- Screening for Eligibility....16
- Approaching by Phone....16
- Storing Data....16
- Outside of the office....16
- **Release of Information**....16

Eligibility Criteria....16
- Inclusion Criteria....16
- Exclusion Criteria....17

Recruitment Methods....17
- Recruitment goals....17
- HMC and UWMC Outpatient Clinic Referrals....17
- **Example email of study recruitment reminder to providers:**....18

Other methods of recruitment....18
- UW TBIMS Newsletter....18
- Websites....19
- Flyers and Posters....19
- Clinic Provider Schedules....19

Pre-screening via Medical Records....20
  - ORCA....20
  - EPIC Hyperspace....20
- Clinic Appointments....20
- Tips for the Clinic....21
- Screening Potential Participants....21
- Summary of Forms Needed for Screening....21

Ineligible at Screening 21
Eligible at Screening 22
Recruitment and Pre-Screening Process..22
Recruitment Letter and Phone Contact 22
Potential Participant Contacts Staff..23
Capacity for Consent 23
Consent Process..23
Consenting In-Person at HMC or UWMC..23
Consent Via Phone..24
Release of Information (ROI) 24
Suicide Protocol..24
Screening Flow Chart..27
Payment Breakdown..28
Declined Compensation 28
Postage and Mailing Supplies 28
Budget Number Barcodes 28
Revolving Fund Account 29
Databases..29
Screening Database 29
Tracking Attempts 29
Status..30
Data Entry 31
Consort Flow Report 32
Outcome Tracking Database 32
Baseline Assessment 32
Baseline Rescreening..33
Data Entry..33
Randomizing a Participant 33
Create a Physical File 33
Notifying Care Manager of Randomization 34
Randomization Flow Chart 35
Outcome Assessments 36
Adding Participant Contact Info 36
Data Entry 36
Quality Assurance..36
TBI-CARE Study Staff 37

TBI Care Study Flow Chart

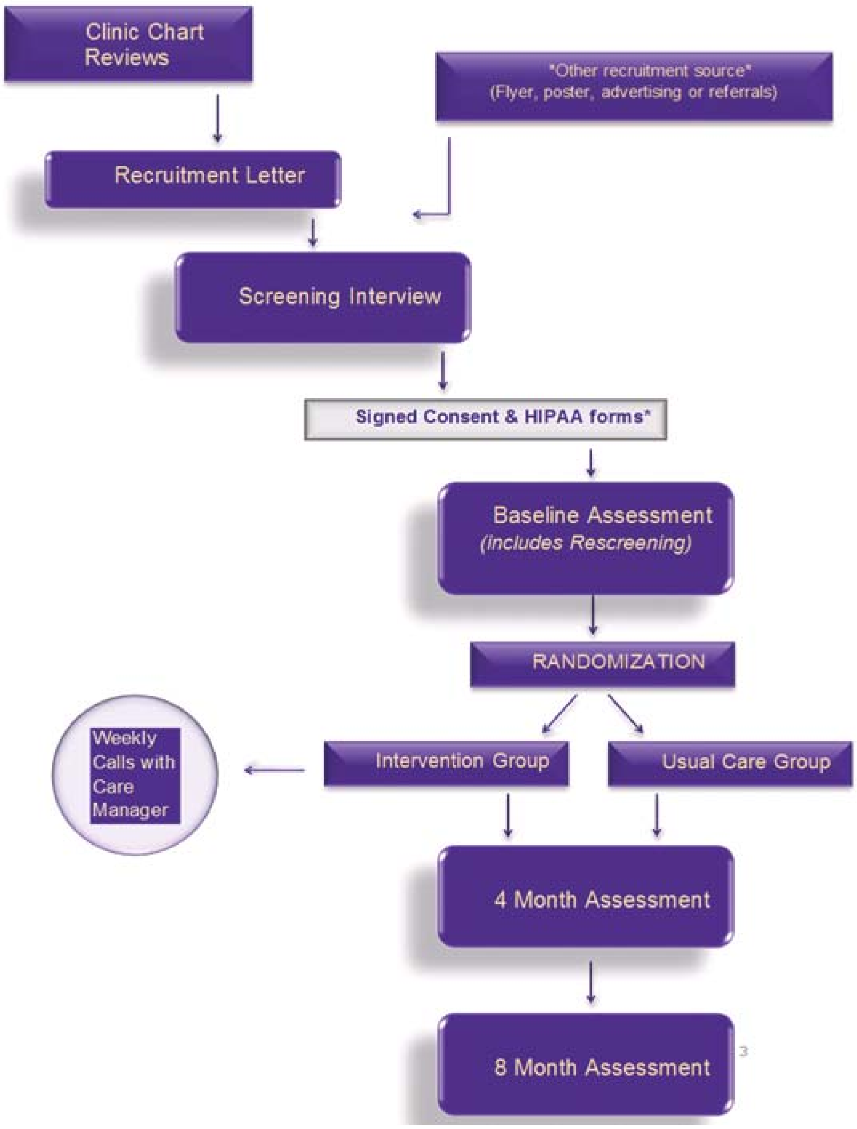

Questions Asked / Answered

Date	Decision
3/21/2020	**RESPONSE TO COVID-19**: Telephone or telehealth visits with the Rehabilitation Clinic provider are considered a visit in which the patient is being "followed" in the same sense of an in-person visit.
03/31/20	Documentation of consent: Waived for main study consent when consent process is by phone or e-mail. HIPAA Authorization: Waived for collecting information from medical record of enrolled participants. Waiver applies only when consent process is by phone or e-mail. Participants will receive a HIPAA authorization form for information but will not be asked to sign it.
05/27/20	TBI CARE exclusion "Presence of severe psychiatric disorder as evidenced by high suicide risk, diagnosis of bipolar disorder with psychotic features or current psychotic disorder within a month of screening". In the event a potential participant has a psychiatric diagnosis (e.g., BP disorder) and a history of a suicide attempt, they may still be eligible as long as they are stable.
05/29/20	Q: My question is whether or not the stutter would be an exclusion? It took her quite a long time to get her new address out. I told her to take her time because it sounded like it exhausted her. She said it was more frustrating. She has a severe stutter and difficult time putting her thoughts into words (that she attributes to her TBI). She also said she would like her twin there to help her when I talk to her again.<br>A: Go ahead and send her the letter and then see how she does with the screening – and if she is still stuttering as badly ask if she wants to do this study? I see they wanted her to see Rehab Psychology but I don't see anything scheduled yet…. If she gets scheduled, that might be the better thing to focus her energy on first, but if she is interested and feels able to engage then we can try.
06/10/20	If someone is indeed unable to communicate over the phone (can't complete the screening) due to aphasia or any communication disorder, the screening form should be coded ineligible due to 6 "Has access to and ability to communicate over the phone" and NOT marked ineligible due to not speaking English.
06/03/2020	Okay to attempt to screen a potential participant who may have a diagnosis of Charcot Marie Tooth disease.
5/22/2020	Q To IRB: Our staff completes follow up data collection by phone and occasionally using Zoom. Recently one of our staff enrolled someone who works for Google and stated he does not want to use the phone or Zoom and insists on using Google Meet, which he insists is more secure than Zoom (although does not appear to be HIPAA compliant). In this specific study's application (TBI Care) we are approved to use phone and we do say video conferencing but in parentheses next to that say (Zoom). Is it okay to collect his baseline data using this other interface?<br>A (Shawn Query, IRB) "… video conference (Zoom)" means, "…video conferencing, for example, Zoom" so I think this is OK without a modification. We don't require that the video conferencing being used is HIPAA compliant, but if you plan to record the sessions, make sure you understand the user agreements and that the company doesn't have outright access to that information, etc. Interesting to me that he's so insistent on using the Google platform.

Date	Decision
04/01/2020	Question about co-morbidities (foot pain following TBI), as long as clinician confirms potential participant "is still actively being followed for TBI issues (gait/spasticity may be related to TBI) then they are eligible.
02/15/2020	Decision to have all participants prior to randomization reviewed by another member of the study team to ensure criteria of being followed by the Rehab TBI Clinic is met. Ideally this will occur prior to administration of the baseline. Rationale: There has shown to be some ambiguity around the meaning of being followed by the clinic.
9/10/2019	In the event that it is not feasible for consent to occur in person, the above procedure will be conducted to obtain verbal consent via phone. In this case we will mail or email out the consent form and HIPAA Authorization but will ask them to keep it for their own records and will not require the signed forms to be returned.
4/18/2019	Do not defer a potential participant during screening if they are not currently in pain (as determined through questioning them using screening form). If they have an upcoming clinic visit they can be rescreened to determine if they are having pain at that time and then be referred to study as appropriate. Rationale: No sense spending time contacting people whose pain may reemerge (e.g., in the next 3 months).
4/16/2019	If during review of medical records, a potential participant is shown as being otherwise eligible for the study but there is no mention of pain, still add to the screening database. Pain can change day to day so add regardless.
3/28/2019	**Clarification of window timeline for data collection:**<br>The 4mo window starts right at 4 months and lasts for 2 weeks post.<br>The 8mo window starts right at 8 months and lasts for 3 weeks post.
2/19/2019	Q: Per Dr. Hoffman, change in procedure – no longer defer potential participants if during screening they are not having pain. Currently we are asking if we may check back in with them in about 9 months. Will start to make ineligible (due to not having pain). They may show up again as potentially eligible in the event they come into the clinic.
1/31/2019	Q: What to do if they mention how often/many times they have seen a provider (in this case it was for counseling). Participant wasn't sure how many and said 12, staff found 5 (looked up due to not knowing discipline of the provider). Do we take self-report (since we wouldn't know this was incorrect unless the appointment was in UW system)?<br>*A: Case by case; confirm with JMH.*
1/30/2019	Q: Eligible? Actively living in Canada (and in school there so not planning to move here anytime soon). There was also this note from Dr. Junn: *Please establish a primary care physician so that we can help manage your symptoms in Canada.*<br>*A: Confirmed not eligible since this person will not be getting primary care from rehab clinic.*
1/30/2019	Charlotte Hoehne Smith, MD approved for eligibility for participants if seen in TBI Clinic (8 North)
1/23/2019	Q: One of our new participants has pain that radiates from the chest into abdomen, so has abdominal pain, it just doesn't originate there. Q: Would #10 - Abdomen - on the Pain Location questionnaire be a yes or no.<br>*Answer: yes*

Date	Decision
1/23/2019	Q: Would “sitting in the tub at home” count as a "water therapy or swimming exercise"? (We had a participant say he does this for pain.)<br>Answer: yes
1/22/2019	Modification approved which includes previously missing CoC language to consent form (now v. 3.2).
1/20/2019	For difficult to reach/no show participants for the intervention will utilize *Difficult to Reach/No-Show Participants – Intervention, SOP*
1/15/2019	Cornell – If someone’s caregiver is also paid, would services be counted on the Cornell #6b-Home aid service? - (including paid homemaker other than primary caregiver). Answer: Regardless of being paid one is still coded as the primary caregiver
1/09/2019	Question of whether someone may be eligible if they have only been seen by an ARNP in the TBI Clinic (rather than a MD)?<br>Answer: If it is Johanne Lewin then yes – though rare, she may see patients completely on her own. If she is following them (and it needs to indicate that in the notes that there is a plan for a next visit) then yes we can include them as she can prescribe, etc.
1/07/2019	SCREENING: When screening if found an upcoming appointment for a NEW patient with a TBI (and seeing a physiatrist or Lewin) but they are NOT a current patient of TBI, add to screening database and defer for 7 days, essentially time enough for the visit note to be reviewed for eligibility. If eligible, mail out recruitment letter at that point and attempt recruitment.
1/07/2019	There may be “some leeway” in the window to complete an outcome as long as the interview has been started, will review by PI on *case-by-case* basis but window to never exceed 4 weeks.
11/12/2018	SCREENING DATABASE: If they screen out on the six-item screener, okay to re-attempt this measure at a later time (if it seems as though they may pass at a later date for example, if they are ‘having a bad day/tired’). This requires some discretion of when to re-test and may be considered on a *case-by-case* basis to confirm with PI.
11/12/2018	SCREENING DATABASE: Participants in pending status are to be moved into ineligible if 1) there is no response to contact after pursuing with due diligence (attempted phone contacts are equal to our approved 9). They can always be returned to the screening database should they have an upcoming TBI appointment.
10/03/2018	Data collection windows for 4- and 8-months post treatment was decided to be a one-month window.<br>From Chuck re SCI-Care:<br>*My guess is that we gave them a one-month window. However, for the four-month outcome we would not have allowed them to assess outcomes prior to four months because the intervention was still be going on. Actually, I don’t think I would have allowed them to assess outcomes prior to eight months either. So, the window was probably within 1 month after the target date.*
09/20/2018	Added Zoom/ screen interface language to consent form as an option for the intervention.
9/10/2018	Outside UWMC record release of Information signature is necessary when treatment for participant’s index injury is outside of UWMC facilities.
08/22/2018	If they screen out on the six-item screener, okay to re-attempt this measure at a later time (if it seems as though they may pass at a later date).

Date	Decision
	Per Jeanne take this on a *case-by-case* basis.
08/12/2018	Decision that if a participant endorses botox, but then botox doesn't appear on the "medications currently taking" list, it shouldn't be entered there.
08/08/2018	Definition of Chronic Pain (used in the grant):<br>Patient reports clinically significant pain, defined as having moderate or higher pain over at least 6 months (defined as an average pain score of 4-10/10 on a 0=no pain to 10=worst pain numeric rating scale).<br>We didn't limit it to number of days because headaches can be less frequent, but still be a problem. Over the past 6 months on average they should have >=4 rating on pain in their body (not just a day or two here and there) or at least 1 headache per month that is severe/disabling (>=7 rating of headache pain), or 4+ with moderate or above pain (>=4 pain rating).
08/08/2018	Question about whether ineligible if participant has participated in prior CC study (in this case SCI Care). Per Jeanne, yes given they meet other inclusion criteria.
07/15/2018	Okay to enroll if they live in Alaska as long as participant sees MD at UW/HMC TBI clinic. No enrollment for anyone living outside of the US.
07/15/2018	Clarified administration of protocol to follow for suicidal ideation ("Emergency Problem Protocol"). Staff will always contact research manager and/ or Investigator for any concern of active and/or passive suicidal ideation (including scoring >1 on the PHQ-9).
07/15/2018	Added language that certain eligibility criteria may be confirmed via conversation with clinical staff.
06/30/2018	Pittsburgh Sleep Index Measure<br>Question: What if there is no bed partner to ask about snoring; can this be self-report?<br>*Answer: As long as they have 'heard that they snore" from a bed partner, self-report is fine (i.e., they don't need to be home for it to be reported by participant*).
06/15/2018	Questionnaire Windows: 14 days before and 14 days after for 4- and 8-month assessments.
06/15/2018	Preference is to enter screening data all at once, or iteratively- if there is no outcome for 'Consent Date', we will list them as 'Pending' in screening database.
06/10/2018	Question about hierarchy for how to code date of injury when more than one has occurred.<br>*Decision to use the most recent injury date as index injury.*
06/10/2018	Question of how much time can pass between screening and enrollment/randomization for the study (i.e., they are in deferral due to a planned surgery, or "not a good time"). Must they be re-screened? What if pain level has changed?<br>*Answer: Handle on a case-by-case basis. Should be rescreened after >2 weeks,*
06/10/2018	If date of injury unknown, okay to use TBIMS protocol (best estimate of year/time of year/using the middle of the month)
06/10/2018	Study's eligibility criteria revised in the following manner: "We will need to limit enrollment to individuals who either have access to a phone or a computer with Wi-Fi" to "We will not enroll individuals who do not have access to a

Date	Decision
	phone.” Rationale: Participants will need access to a phone for the outcome questionnaires, so internet access alone would not suffice.
06/03/2018	Response key will be sent out to participants to help with administration of follow-up questionnaires.
05/22/2018	Received IRB approval to start recruiting! Will need to get roughly 5 participants/month.
05/16/2018	Question about the recommended strategies to describe TBI severity way to document TBI for this population (as a reminder the study will be recruiting participants from the UW and HMC TBI clinics for those w/ a mild/complicated – severe TBI and >6 months out from the date of their injury. (In addition to TBI Clinic physician referrals, we will be recruiting via mailing to those who have upcoming visits as well as people may see a flier at the clinic and refer themselves.) Since they will all have rehab clinic notes the decision was to pull from the summary statement about the injury.
05/16/2018	Q: How to code if not labeled as in the chart as mild-severe?<br>A: Always refer to treating clinician (physiatrist) to ask and if difficult to obtain, refer to Jeanne.

**Study Title / Number:**	**Formal:** ***The Effectiveness of Collaborative Care versus Usual Care for Pain after Traumatic Brain Injury***<br>**Informal:** ***TBI Care***<br>**ID: STUDY00003847**<br>**Approved through 12/21/2023**

Interaction	Description	Submission Date	Approval Date
Initial Application	Application and Screening Response 1, 2, and 3 Conditional Approval (MRSA) Approval Period: 05/14/18 – 05/07/2019	04/24/18	05/14/18
Approval Study#00003847	Full approval	05/16/18	05/22/18
Modification 1 MOD00002201	Modification to Zipline protocol, study forms updated, resource list to use for UC group updated, brochure and flyer added, consent form updated.	05/24/18	06/19/18
Modification 2 MOD00002457	Modification to Zipline protocol, CoC updated; Zoom added as an option for the intervention, screening form added, consent form updated.<br><br>Screening Form: 1) Clarified headache pain rating, 2) exclusion criteria and 3) corrected skip pattern<br>Headache Pain Rating<br>Previous verbiage, page 1 under heading labeled PAIN:<br>5. Have you had pain, which may include headache, in the last 6 months?<br>☐ 0 If No: They are not eligible at this time. Proceed to DEMOGRAPHICS at the end.<br>☐ 1 If YES, continue on.<br>6. Please rate your average pain (including headache) in the last 6 months/since your injury on a scale of 1 to 10, with 1 being the least pain and 10 being the worst pain imaginable _______<br>If average pain number is > or = to 4, continue to question #7; If average pain number is <4, they are not eligible at this time. Proceed to DEMOGRAPHICS at end.<br>Revised: 5. Have you had chronic pain, which may include headache, in the last 6 months? ☐ 0 If No: They are not eligible at this time. Proceed to DEMOGRAPHICS at the end. ☐ 1 If YES, continue.	07/14/18	07/26/18

Interaction	Description	Submission Date	Approval Date
	6. If pain only includes headache: How often do you have headaches? If <4 times per month, average pain rating needs to be 7 or higher<br>7. When you have headaches, what is your average pain rating with 1 being the least amount of pain and 10 being the worst pain imaginable? _______________ If pain is not headache related, continue:<br>8. Over the past 6 months, can you tell me on average how severe your pain is on a 1 to 10 scale with 1 being the least amount of pain and 10 being the worst pain imaginable?<br>If average pain number is > or = to 4 (or >7 if headaches is <4 times per month), continue to question #8; if average pain number is <4, they are not eligible at this time.<br>2) Clarification of exclusion criteria, Page 3 Previous verbiage: "Patient does not have diagnosis of bipolar disorder with psychotic features or current psychotic disorder"<br>Revised: "Presence of psychiatric disorder with psychotic features (with delusions and/or hallucinations) within the past month of screening"<br>3) Corrected skip pattern (page 3). Should not be excluded based on psychiatric history<br>Consent Form:<br>Page 3 Changed use of word "call" to the more inclusive (of video or calling) "contact" where appropriate<br>Page 4, 5 Added verbiage about using ZOOM (HIPAA compliant) videoconferencing as an option. While we don't anticipate many participants randomized to TBI-Care intervention with the Collaborative Care Manager (CCM) will choose to use video conferencing over telephone, we would like to be able to provide that option.<br>Zipline Protocol:<br>Page 8 and 12 clarification of exclusion criteria: Removed: "Diagnosis of bipolar disorder with psychotic features or current psychotic disorder." Revised to: "Presence of psychiatric disorder with psychotic features (with delusions and/or hallucinations) within the past month of screening." Page 12 - Clarification of protocol of phone contact made post-recruitment letter being sent (as needed only). Contact attempts •		

Interaction	Description	Submission Date	Approval Date
	Up to 3 attempts during normal business hours (at different times of the day) • Up to 3 attempts during weekday evenings (on different days of the week) • Up to 3 attempts during weekends<br>Page 17, 18, 26 - Added using ZOOM (HIPAA compliant) videoconferencing as an option.<br>Page 23 Removed: "Patient does not have diagnosis of bipolar disorder with psychotic features or current psychotic disorder"<br>Revised to: "Presence of psychiatric disorder with psychotic features (with delusions and/or hallucinations) within the past month of screening."		
Modification 3 MOD00003098	Modification to Zipline protocol, screening form revised, consent form updated.	11/06/18	11/14/18
RNI 1 (MOD4)	Protocol violation: Subject randomized who saw Dr. Dasher in clinic (Psych) rather than TBI doc. Confirmed with Galen not necessary to submit to IRB as no increase in risk.	Dropped by HSD	Not submitted
Modification 5 MOD00003517	Uploaded the Certificate of Confidentiality for TBI Care - site specific project for this cycle for the TBI Model System - Application # STUDY00001788; Certificate of Confidentiality (uploaded); MH-DOE-99-002B; Issued 09/18/2018; Date of Exp. 12/31/2023.	1/19/2019	1/24/2019
Modification 6 MODCR00001678	First year continuing review. Approval from 4/15/2019 to 4/14/2020.	04/07/19	04/15/19
Modification 7 MOD00005517	Updated research staff (added Meghan Gill to our informed consent form document).	01/02/20	01/06/20
Modification 8 MODCR00000313	Second year continuing review. Approval period is 3/25/2020 through 3/24/2021.	03/05/20	03/25/20
Modification 9 MOD00006240	Approved to consent remotely due to COVID-19. NOTE: While IRB approval for this modification and continuing review has been granted, the University is requiring a temporary halt of some research activities that involve in-person interaction with participants.<br>This temporary halt is effective March 23rd through at least May 1st, 2020 and may be extended further. For additional information about the halt and the current effective date, see the HSD website: https://www.washington.edu/research/hsd/covid-19/. Requested waiver of written documentation	03/30/20	03/31/20

Interaction	Description	Submission Date	Approval Date
	for consent and HIPAA authorization for participants enrolled by phone/email.		
CR00004223	Third year continuing review. Approval period is 2/19/2021 to 2/18/2022.	2/12/21	2/19/21
Modification 10 MOD00011664	Requested waiver to share contact info from screening database with ETIPS – STUDY00005686 Efficacy of a Telehealth Pain Self-Management Intervention in Employed Adults with Physical Disability: A Randomized Controlled Trial. PI: Dawn Ehde	11/29/21	12/08/21
MOD00012002	Consent waived for Emergency Department Information Exchange (EDIE) records review.	1/11/21	1/14/21
CR00005578	Fourth year continuing review. New approval period 1/14/2022 to 1/13/2023.	1/12/22	1/14/22
CR00006816	Fifth year continuing review. New approval period 12/22/2022 to 12/21/2023.	12/12/22	12/22/22

*Continuing reviews had been referred to as “modifications” until 2021.

STUDY DESCRIPTION

We plan to enroll approximately 158 adults with mild, moderate and severe TBI who are current patients (within the last 12 months) of the outpatient rehabilitation TBI clinic from either Harborview or the University of Washington Medical Center. We will over enroll the number of participants proposed: 158 recruited to complete 126 follow ups. Eligible participants will be recruited, consented and randomized into one of two groups: Usual Care (UC) or Collaborative Care (CC). Randomization will be conducted by one of our research team members who does not complete study assessments to ensure research staff collecting data remain blinded to assignment.

TREATMENT CONDITIONS

Usual Care group

Participants assigned to usual care will be informed by the CCM or another un-blinded member of our research team of their assignment and encourage them to consult with their TBI or primary care provider with any concerns. Study personnel will not make any attempts to influence usual care participants’ pain management.

Because this is an effectiveness study, treatment decisions for UC participants will be left to the primary provider and may or may not include pharmacologic management, counseling, and referral to specialty services or other local resources.

Collaborative Care Group

The Collaborative Care (CC) intervention will involve up to 12 sessions (45-60 minutes long) with a Collaborative Care Manager (CCM) over 16 weeks of treatment. Weekly sessions with CCM could take place via phone, video, or in-person.

Collaborative Care - Overview

Collaborative Care (CC) is a systematic and integrated approach to improving the delivery and utilization of effective treatments for chronic pain. The care will be delivered through an interdisciplinary team, organized around a Collaborative Care Manager (CCM) who guides the participant through various aspects of care. The CCM will offer all participants care management, collaborative medical management, and psychosocial treatment. If the participant declines to receive any of the components of the intervention, she/he may still choose to participate in the remaining components.

Data Analysis Methods

Data will be collected on paper forms and entered into a specially-designed Access database that incorporates data quality checks. Reports monitoring the progress of the study will be generated monthly to help the investigators identify areas such as enrollment or follow-up rates that might need specific attention. Analyses will be performed using SPSS and SAS. The analysis plan will conform to the intent-to-treat principle: all randomized cases will be included based on their assigned treatment group regardless of actual treatment received. Those not assessed at the end of treatment will be considered to have missing values. If the missingness mechanism is such that it allows for imputation, we will use multiple imputation.90

Prior to the analyzing for the effect of CC, we will compare the groups on pre-randomization characteristics using Mann-Whitney tests or Fisher's exact tests depending on the type of variable. For the primary analysis of Aim 1, we will compare the CC group to the UC group on pain interference at the end of treatment (the 4-month outcome). We will use a linear mixed effects model with random effects for intercept and slope and controlling for stratification variables and any relevant pre-randomization imbalances. The parameter of interest is the interaction of treatment by time. A 2-sided significance level of 0.05 will be used.

We plan to analyze Aim 2 hypotheses using similar mixed models for outcomes measured at baseline and t-tests for outcomes without baseline assessment. For assessing secondary hypotheses regarding maintenance of treatment effect, we will also use mixed models including all three time points and considering time as categorical to allow for diminished effect after treatment ends.

HIPAA General Guidelines and Confidentiality

The Health Insurance Portability and Accountability Act (HIPAA) is a federal law designed to protect the privacy of a patient's health information. All employees must complete the HIPAA training provided online through the University of Washington. This can be found at http://www.washington.edu/research/hsd/faq_hipaa.html
Additional study procedures designed to protect the privacy of health information are listed below.

Screening for Eligibility

Employees are only allowed to access medical records of participants or potential participants for the study. When accessing a patient’s record to determine if they are eligible, the patient’s HMC or UWMC ID must be documented in the screening database.

Approaching by Phone

Unless the RA knows that the participant is the only person with access to phone messages, only state your name and that you are calling from a research study at the University of Washington and Harborview Medical Center. Do not disclose the name of the study, and or the nature of the study (i.e. TBI, pain) when leaving a phone message.

Storing Data

All identifying information is removed and stored separately from the study data. An identification number is used to link the information. All study data is stored in locked filing cabinets on a locked floor of NJB.

Outside of the office

It is permissible to speak about a participant when away from the office, but no identifying information may be disclosed. The HIPAA training describes what identifiable information is, however, if the participant received a lot of media attention, anything mentioned about the participant could be identifiable.

Release of Information

If it is necessary to obtain PHI from another provider or research study, a Release of Information (ROI) form is required. It is also necessary to use a ROI form when participants would like study staff to release information about their study participation to an outside provider (see Release of Information in the Screening Potential Participants section).

Eligibility Criteria

To be eligible for the TBI-CARE study, potential participants must meet inclusion criteria.

Inclusion Criteria

- Definitive diagnosis of mild, moderate or severe *TBI based on medical chart review;
- At least 6 months post injury;
- Patient has an appointment in the HMC or UWMC Department of Rehabilitation Medicine TBI clinics or has been seen by TBI providers within the last 12 months;
    - Participant does not need to be seen for primary care to be eligible for study, consulting care at the UW or HMC rehab outpatient clinics is sufficient but must be followed by the TBI provider.
- Patient reports clinically significant pain, defined as having moderate or higher pain over the last 6 months (defined as an average pain score of 4-10/10 on a 0=no pain to 10=worst pain numeric rating scale);
- Patient is “somewhat” or “very” willing to accept additional help with their pain as asked during screening;
- Has access to and ability to communicate over the phone;
- Aged > or = to 18 years of age;

- Provides verbal or written informed consent to join the research study.
- Able to speak and understand English sufficiently to complete the screening and outcome measures, and to participate in a verbally based treatment program (we will track non-enrollment due to other language to determine common languages).
    - There are no cut-and-dried criteria for this determination, and the information verifying English fluency/ lack thereof may come from different sources, including study personnel after interacting with the participant on the phone, referring clinicians, or medical records.
    - It may be helpful to address the following questions to a reliable source:
        - How difficult is it for the person hold a conversation in English?
        - How difficult is it for the person to understand and follow television programs, movies or listen to the radio in English?
    - Uncertain cases should be brought to the team for discussion.

***TBI defined as by the TBI Model System**

Exclusion Criteria

- Answers more than one incorrect response on the 6-Item Screener;
- Terminal illness or pain associated with cancer diagnosis;
- Major surgery anticipated during study period (approximately 8 months);
- Presence of psychiatric disorder with psychotic features (with delusions and/or hallucinations) within the past month of screening;
- We will not enroll individuals who are not able to provide consent for themselves.
- We will not enroll individuals who are in law enforcement custody or who are taken into custody during the study period due to federal restrictions on inclusion of prisoners in research.

Recruitment Methods

Recruitment for TBI-CARE participants will come from multiple sources. The primary focus of recruitment will be based on a review of the upcoming clinic schedules of TBI providers and a pre-screen of potential participants who are scheduled to come to UWMC and HMC outpatient clinics. Prescreening for patients coming into the clinic with a diagnosis of TBI will be sent a recruitment letter, followed by a phone call approximately two weeks later.

Recruitment goals

The total enrollment goal for the TBI-CARE study is 158 participants over a four-year period. In order to reach our participant goal of 158, approximately 40 participants must be enrolled every year. A recruitment report will be generated and the enrollment rate will be monitored on a regular basis. Any concerns or unusual attrition rates should be discussed by the team.

HMC and UWMC Outpatient Clinic Referrals

Participants may be referred to the TBI-CARE study by TBI clinicians, PT/OT staff, or other patient-centered clinic staff at Harborview and UW Medical Centers. Clinicians acting as recruitment intermediaries may refer on the basis of clinical judgment or as response to emails about the study sent out to providers. Referrals from UW and HMC clinicians may be made directly to TBI-CARE research coordinator by providing the name, hospital ID, and phone number of the patient wishing to be contacted by study staff. Outpatient staff may also provide

study flyers to refer potential participants to contact coordinator by phone or by email. If a referral is sent to study staff by a clinician this referral is prioritized as typically the clinician will have just seen them in clinic and spoke with them about the study to determine their interest while there. Once contacted, or if unable to contact, an introductory letter and study flyer must be mailed to the potential participant.

Example email of study recruitment reminder to providers:

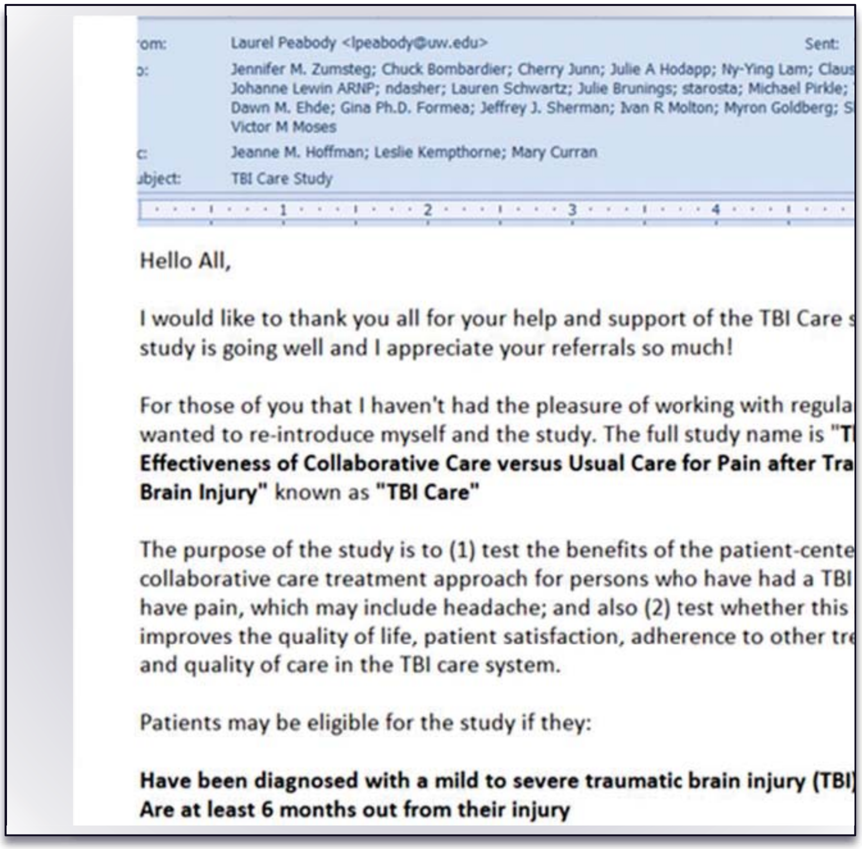

rom: Laurel Peabody <lpeabody@uw.edu> Sent:
o: Jennifer M. Zumsteg; Chuck Bombardier; Cherry Junn; Julie A Hodapp; Ny-Ying Lam; Claus
Johanne Lewin ARNP; ndasher; Lauren Schwartz; Julie Brunings; starosta; Michael Pirkle;
Dawn M. Ehde; Gina Ph.D. Formea; Jeffrey J. Sherman; Ivan R Molton; Myron Goldberg; S
Victor M Moses
c: Jeanne M. Hoffman; Leslie Kempthorne; Mary Curran
ubject: TBI Care Study

Hello All,

I would like to thank you all for your help and support of the TBI Care s
study is going well and I appreciate your referrals so much!

For those of you that I haven't had the pleasure of working with regula
wanted to re-introduce myself and the study. The full study name is "**T**
Effectiveness of Collaborative Care versus Usual Care for Pain after Tra
Brain Injury" known as "**TBI Care**"

The purpose of the study is to (1) test the benefits of the patient-cente
collaborative care treatment approach for persons who have had a TBI
have pain, which may include headache; and also (2) test whether this
improves the quality of life, patient satisfaction, adherence to other tr
and quality of care in the TBI care system.

Patients may be eligible for the study if they:

Have been diagnosed with a mild to severe traumatic brain injury (TBI)
Are at least 6 months out from their injury

Other methods of recruitment

UW TBIMS Newsletter

A reoccurring blurb about TBI-CARE is routinely featured in the UW TBI Model System's quarterly newsletter, *TBI Updates*. The information includes contacts for the research coordinator for interested participants.

Websites

The TBI-CARE study is listed on the following websites for participants seeking to join research studies:

Department of Rehabilitation Medicine
http://rehab.washington.edu/research/studies/TBI.asp#care
UW TBI Model System
https://tbi.washington.edu/tbims-research-studies/
University of Washington Health Sciences—Research Studies Seeking Volunteers*
http://www.medical.washington.edu/studies/
ClinicalTrials.gov
https://www.clinicaltrials.gov/ct2/show/record/NCT03523923?term=Collaborative+Care+versus+Usual+Care+Telephone+Brain+Injury&draw=2&rank=1

Flyers and Posters

IRB approved brochures and flyers are distributed throughout HMC and UWMC. There are general placeholders for flyers in the 8 South outpatient rehab clinic at UWMC, and at the CORP waiting area at HMC. TBI-CARE recruitment posters are to be posted in the CORP exam and consult rooms and in the UWMC rehab clinic exam rooms. Passive recruitment methods, such as posters or flyers, should not be relied upon as the main source of recruitment leads.

Clinic Provider Schedules

A review of the provider clinic schedules (approximately 1 month in advance) will be maintained in order to find potential participants for the study. Providers who have allowed access to their clinic schedules include:

Jennifer Zumsteg, MD	zumsteg@uw.edu	Harborview Medical Center CORPS Clinic – 4th Floor Maleng Building
Cherry Junn, MD	cjp42@uw.edu	Harborview Medical Center CORPS Clinic – 4th Floor Maleng Building
Julie Hodapp, MD	hodapp@uw.edu	Harborview Medical Center CORPS Clinic – 4th Floor Maleng Building
Natasha Mehta, MD	mehtaj@uw.edu	Harborview Medical Center CORPS Clinic – 4th Floor Maleng Building
Deborah Crane, MD	dacrane@uw.edu	Harborview Medical Center CORPS Clinic – 4th Floor Maleng Building
Charlotte Hoehne Smith, MD		University of Washington Medical Center - 8 South rehab clinic

Lesley Abraham, MD	labrah@uw.edu	University of Washington Medical Center - 8 South rehab clinic
Johanne Lewin, ARNP	lewinj@uw.edu	Harborview Medical Center CORPS Clinic – 4th Floor Maleng Building

An introductory letter explaining the study will be sent to potential participants. Potential participants will be able to contact study staff if they are interested in learning more about the research, or if they wish to be removed from the recruitment list.

Pre-screening via Medical Records

While viewing provider schedules for upcoming clinic appointments, the reviewer should - review the following minimum eligibility criteria in the medical record:

- Traumatic Brain Injury
- At least 18 years old
- 6 months post injury
- English Speaking

Exclusionary factors that might be discovered during preliminary medical record review:

- Non-traumatic brain injury
- Non-English speaking (requires interpreter services)
- Diagnosis or recent treatment for Bipolar Disorder, Psychotic Disorder, Schizophrenia or Schizoaffective Disorder
- Major upcoming surgeries (scheduled within 8 months of review)
- Cognitive or communication deficits, which may prevent participation in a verbally-based treatment program (such as Dementia, Alzheimer's or other degenerative condition)

Provider schedules can be viewed in both EPIC Hyperspace and ORCA Powerchart systems.

ORCA

Practitioners in UW Medicine currently use ORCA to edit, sign, and forward transcribed documents and for entry of new notes using several direct entry tools. It is also used for results review, for medication profile review and as the inpatient pharmacy system. Patient medical records can be viewed in ORCA during the pre-screening process for the TBI-CARE study by authorized study staff.

EPIC Hyperspace

EPIC allows for viewing multiple schedules at once and often provides extra clinic notes regarding patients in the schedule viewer. Training for EPIC is available by appointment and upcoming trainings can be found at: https://hmc.uwmedicine.org/BU/AACSBusiness/Pages/TrainingCalendar.aspx?CalendarDate=7%2F20%2F2013

Clinic Appointments

Clinic appointments can be utilized for in-person recruitment/study introduction by research staff members. The research coordinator will conduct a review of the schedule for all TBI providers in

the UWMC/HMC outpatient clinics 3 weeks in advance and also on a weekly basis. If they are available to attend the clinic appointments, then an email notification listing times and names of potential participants should be sent to each provider at least one full day in advance.

Clinic appointments can be used to introduce the study to potential participants who have not been reachable by phone. Additionally, some potential participants may request study staff to conduct interviews or collect signed consent forms during the clinic appointments.

Tips for the Clinic

Each clinic provider has specific preferences for how to go about utilizing his/her clinic schedules for recruitment or follow-up. Some providers prefer to consult with the research member before (s)he sees the patient. Overall, sending weekly notifications about which patients will be approached during the clinic schedule should be done for all providers and each provider will develop a rhythm with research staff over time. Nurses and front desk staff are also key players and can help coordinate in-clinic visits.

Screening Potential Participants

All potential participants should be contacted approximately two weeks after a letter and flyer are sent in the mail. If a referral is made by a clinician or other clinic staff member, a letter and study flyer should be sent prior to calling the potential participant on the telephone.

The study **Telephone Approach Script** should be read to potential participants to inform them about the study, and their potential role as participants. If the potential participant is interested in the study after hearing the information and having had an opportunity to ask questions, the **Screening Form** should be used to determine eligibility. If the potential participant is unable to perform the screening interview immediately after the study intro, the examiner should schedule a time to contact the potential participant again at a later time.

For individuals who do not want to join a research study, the examiner should attempt to complete a demographics questionnaire nonetheless.

At least three but not more than 10 attempts should be made to contact a potential participant. Attempts should be made on different days of the week, and different times of day. If a potential participant does not return contact, or cannot be contacted, their status can be “”deferred” in the screening database.

Summary of Forms Needed for Screening

Approved documents are located on the Hemingway server: T:\TBI Care\Recruitment

1. Telephone Approach Script
2. Screening form

See screening flow chart

Ineligible at Screening

Discuss rescreening past potential participants with the team on a case-by-case basis. Data for ineligible participants must be entered into the screening database once a medical chart is clicked on. If a patient chart is opened, their information (first and last name, hospital ID number, and date of birth must be entered into the screening database. If they are found to be ineligible after opening their chart, select the reason for ineligibility in the dropdown menu.

Eligible at Screening

For participants who are eligible after they have completed the screening process, a packet of information should be provided/ sent including:

1. Intro letter
2. Study Consent Form (signed by research assistant)
    - Write-in the participant's full name and highlight areas where the participant must sign/date. Also place "SIGN HERE" tabs next to the signature line.
3. HIPAA Authorization Form (with ROI information, if relevant)
    - Write-in the participant's full name and highlight areas where the participant must sign/date. Also place "SIGN HERE" tabs next to the signature line.
    - Under section **F. GIVING PERMISSION**, highlight the "Behavioral or mental health/illness, including psychotherapy notes" section and place an "INITIAL HERE" tab next to the initial line. Participants must give us express permission to access these records by initialing on the adjacent line.
4. Return Address Envelope (with barcode label and "Attn: Research Coordinator" label)
5. The packet should be sent in a large manila mailing envelope (with UW return address label).

Recruitment and Pre-Screening Process

All participants will be recruited through the two UW Medicine outpatient Traumatic Brain Injury Rehabilitation Clinics at Harborview Medical Center (HMC) and University of Washington Medical Center (UWMC) of which the majority will come from our research staff prescreening upcoming appointments. Participants will be recruited by age, gender and race in the proportion they are represented in the sample of eligible participants. Within the clinic, there are several ways we will be recruiting and screening for this study.

The first will involve pre-screening medical records for upcoming clinical visits for which case we have an IRB approved HIPAA waiver in place. Also, interested individuals may contact our team after viewing a study brochure or flyer posted in the TBI clinic at HMC or UWMC. We have IRB approval to obtain non-written consent to ask eligibility questions over the phone.

All potential participants are assigned a screening ID number. The participant will keep the same screening ID number if enrolled in the study but will receive another study number once they have been randomized to a treatment group.

Recruitment Letter and Phone Contact

Those deemed eligible based on medical record review will then be mailed a letter letting them know that the study is enrolling and inviting them to contact our research staff to learn more and/or be screened for inclusion. This letter also lets them know that they may be contacted and/or approached in the TBI clinic to see if they are interested in learning more about the study. The letter will be accompanied by the study brochure that provides more detailed information.

Phone Contact Attempts May Include:

- Up to 3 attempts during normal business hours (at different times of the day)
- Up to 3 attempts during weekday evenings (on different days of the week)

- Up to 3 attempts during weekends

Potential Participant Contacts Staff

Once eligibility has been confirmed through the screening process, a research staff member may arrange a time to conduct the informed consent process via phone. The research staff member will then either send out a self-addressed, stamped envelope along with two copies of the consent form, the HIPAA authorization form and a cover letter or depending on the preference of the potential participant, an email with the same information. (For those who wish to do the consent process via email, it will be advised to them that email security cannot be guaranteed). The approach letter will specify that although participants may review the consent form in advance, they should not complete the forms until they have reviewed the forms with research staff at the scheduled informed consent session via phone.

Capacity for Consent

Ensuring capacity for consent is handled during the screening process by administration of the 6-Item Screener. Once a potential participant is deemed able to provide consent (not missing more than one response on 6-Item Screener) our research staff member will review of the informed consent form (ICF) approved by the UW IRB. Potential participants will be asked to repeat back understanding of this material as necessary.

Consent Process

Informed consent is an ongoing process. When interacting with participants it is necessary to continue to outline what their participation involves, answer questions, and reiterate the voluntary nature of participation as necessary. Research staff will obtain informed consent from research participants after the screening process but prior to commencement of any further study procedures. The informed consent process may take place at HMC or UWMC, or via phone with our research staff.

Consenting In-Person at HMC or UWMC

Once a potential participant is deemed able to provide consent (not missing more than one question on the 6-Item Screener), our research staff member will review with them informed consent form (ICF) approved by the UW IRB. Potential participants will be asked to repeat back understanding of this material as necessary. Our research staff is trained to ensure competency to discuss informed consent and strategies to ensure there is no coercion. Potential participants will be fully informed of all risks and benefits prior to giving their written informed consent and prior to enrollment in the study and will be provided with as much time as needed to review the ICF and ask the research staff member questions about the ICF, their rights as participants, and participation in the study. If during the course of this contact the potential participant has questions that cannot be addressed by research staff, one of the study investigators or the research manager (depending on the nature of the questions) will follow up with them to ensure all questions are addressed.

If a potential participant is willing at this point, they will then be asked to sign and date the ICF, or in the event the participant is providing verbal consent they may sign and keep for their own

records. Research staff will also date and sign the ICF. Interested participants will also sign or verbally consent to a HIPAA authorization form allowing staff to collect information from UWMC medical records as well as from outside providers as needed. Staff may also ask participants to sign a standard UWMC authorization form to release/disclose protected health information (PHI) (‘release of information form’). Completion of this form would permit the CCM to contact designated health providers of the participant to provide recommendations for care discussed during study treatment, e.g. prescription of antidepressants. The PHI is not a required document and will only be requested in the case that the CCM would benefit from contacting an out-of-network physician. However, participants must sign the HIPAA authorization form.

Consent Via Phone

At the scheduled phone contact, a research staff member will follow the informed consent procedures as described above.

Unable to Contact Participants

Participants that are unable to be contacted via phone, email or text will be sent a letter (requesting the participant contact our research staff).

Participants who are eligible after the screening interview will be sent an informed consent form and HIPAA Authorization form through the mail. Any questions will be addressed by study staff prior to sending the forms. Potential participants may also call study staff with any questions or concerns before signing and returning the forms. Consent may also be obtained if the potential participant is approached in person, or during a clinic visit. Once the signed forms are returned to study staff, from this point forward, the participant is enrolled in the study.

Release of Information (ROI)

During the screening interview, the examiner will ask the participant about health care providers seen outside of the UW Medicine system. If the participant does seek outside health care treatment, then we will want to obtain Release of Information (ROI) Authorization, especially for those providers who may manage medications for pain, mood, depression, anxiety, PTSD or sleep.

The name of health care organization(s) or provider(s) must be written in the space provided on the front of the HIPAA Authorization Form, along with phone numbers (if available). The information should also be recorded on the Screening Form interview. Notes about any other providers should also be recorded in the Screening Comments section of the screening database for each participant.

Suicide Protocol

The PHQ9 measure is a component of the screening interview and the baseline rescreening, which may trigger our Suicide Protocol.

Both the screening and the baseline rescreening contain explicit instructions and guidelines for following the suicide protocol.

Summary of Suicide Protocol

1. Research staff will first check to see if the participant has had thoughts of wanting to harm themselves or if they had thoughts of being better off dead.

2. When a participant endorses thoughts of death and self-harm in the past two weeks during the PHQ9 measure, the interviewer will ask if the participant has discussed this with a health care provider. If the participant's doctor knows about suicidal ideation, ask about plans developed to stay safe between the participant and doctor. As a follow-up, ask if the participant is getting enough help to manage these thoughts or feelings. If the participant is getting enough help, then offer the participant the number for the crisis line(s). Document responses on the screening form. If the participant's doctor does not know about the participant's suicidal ideation or thoughts of self-harm, then the study PI and/or clinician will need to call the participant.
3. Regardless of how the participant responds to the above questions the interviewer will give the participant local emergency contact numbers they can call for help:
    - King County Crisis Line: 206-461-3222
    - Snohomish County Crisis Line: 425-258-4357
    - Pierce County Crisis Line: 253-396-5180
    - Hopeline (National Crisis Line): 1-800-SUICIDE (784-2433)
    - Domestic Abuse Hotline: (206) 522-9472

4. The interviewer will then ask the follow-up question: "*Do you feel that these thoughts are a problem for you, or something you might act on*?"

    If "yes," the interviewer will ask the participant: "*Do you have a plan on how you would do it? I am not a clinician, but we want a clinician on the study to talk with anyone who tells us they have been feeling this way recently. I would like a study clinician to give you a call today. Would that be okay?"*

    If the participant is imminent for suicide and or has a plan, make sure the participant agrees to not act on these feelings and to talk to the study physician. Depending on the severity of thoughts, it is imperative to stay on the line with the participant and page a study clinician on another line, or have another research member page/call a study clinician.

    If the participant refuses to talk to a study clinician, an agreement (contract) must be made with the participant to not act on their feelings and to speak with their doctor. Research staff is to immediately page a study clinician (preferably Drs. Hoffman, Fann or Collaborative Care Manager) and explain a participant refuses to speak with someone from our study. **In extreme cases, when a participant does not want to speak to a clinician, but is imminent for suicide, (or other violent/harmful behavior) the interviewer should call 911 for an immediate intervention.**

5. After the interview, for all participants endorsing thoughts of death and/or self-harm, the interviewer will contact a study clinician (Jeanne Hoffman, Jesse Fann, or Collaborative Care Manager) and the Research Manager, via phone or pager, leaving the following information:

    - Participant's name
    - PHQ-9 Items endorsed
    - If participant has discussed this with his/her health care provider.
    - If the participant has a regular care provider.
    - Participant's response to the follow-up questions.
    - Participant's phone number and location.

- Any other comments made by the participant.

Clinicians must provide a written summary or update about the SI follow-up call, which will be printed and placed in the participant's file.

For participants who have already spoken with a study investigator and are already receiving help from a doctor or mental health professional, a PI may not need to contact the participant again but will be determined by the PI on a case-by-case basis. Research staff will e-mail the Research Manager and the appropriate PI with an update on the participant.

Screening Flow Chart

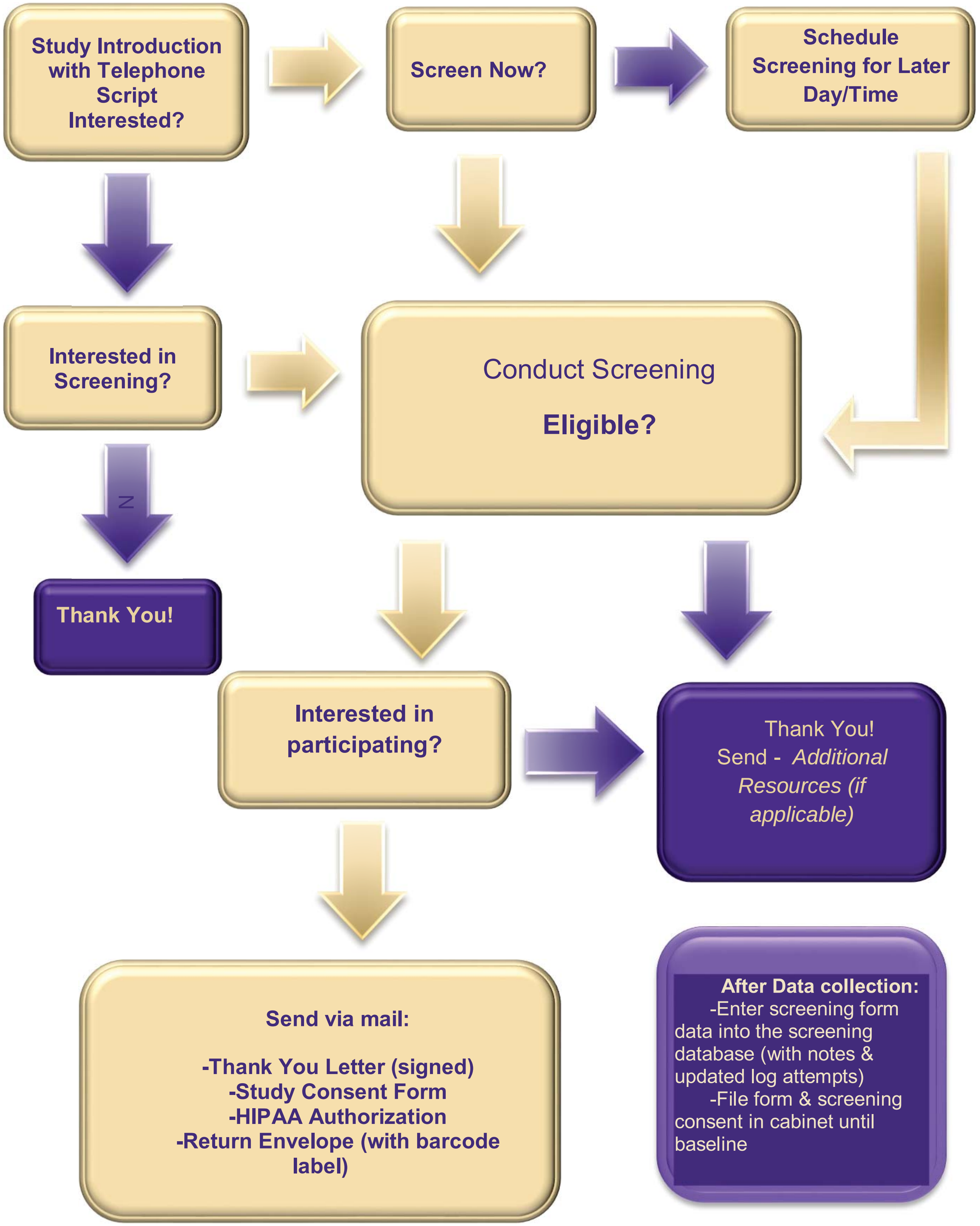

Participant Compensation
Participants are compensated up to $50 total for their time and participation in the study. Participants may choose to decline payment for the study or individual interviews, in which case, a thank you letter is sent mentioning that declining payment for an interview does not invalidate payment for future interviews with the study.

Payment Breakdown

Assessment	Payment	Approximate Time
Baseline Interview	$10.00	30-45 minutes
4 Month Assessment	$20.00	25-35 minutes
8 Month Assessment	$20.00	25-35 minutes

Declined Compensation

If a participant chooses to decline payment for interviews, note: “Declined Compensation” in the TBIMS check register with a note field of the corresponding database.

Postage and Mailing Supplies

If sending mail from Harborview Medical Center, no postage is required for outgoing mail, however, envelopes must have UW/HMC mailing return addresses in order to be sent. If sending mail from UW, budget barcode labels are required for outgoing mail.

Budget Number Barcodes

The UW uses a barcode system for charging postage to a budget in place of stamps for outgoing or return mail. Barcode stickers can be ordered from Creative Communications by sending an email to **barcode@u.washington.edu**. Barcode stickers are to be placed in the upper *left* hand corner of an envelope.

When ordering barcode labels, include the following information:

- Your Name
- Department name
- Box number
- Email address
- Phone number
- Quantity of label sheets (80 labels per sheet)
- Budget number and budget name *(budget numbers are changed annually around September—contact Leslie Kempthorne for updated budget numbers)*
- P.I. or person in charge of reconciling the budget

Labels are delivered to the contact person in each department, and then can be distributed to faculty or staff. Barcode labels are produced at Mailing Services and are supplied at no cost to University departments.

Harborview does not use the barcode system for outgoing mail postage.

Revolving Fund Account

A revolving fund account is an advance of funds to an individual custodian on behalf of a department. Revolving fund accounts are mainly used for small dollar authorized purchases and paying research participants. As purchases are made, departments must request reimbursement in order to replenish the account. Records of each payment to research participants via revolving fund **are** kept and reported to the Financial Services Tax Desk at the end of each year, as applicable. Our revolving fund account is a checking account to be used for participant payments and reimbursements.

The UW TBI Model System uses the Central Fund account held by the Dept of Rehab Medicine and overseen by Leslie, Erica and fiscal specialist, Sandra Fong.
Staff are responsible for issuing payments from the account and updating the account register stored on Hemingway here: T:\TBIMS 2017-2022\Admin\Budget\Central Fund Starting Oct 2019

Authorized Account Signers:

- Erica Wasmund
- Leslie Kempthorne
- Jeanne Hoffman

Databases

Screening and the tracking databases for the TBI-CARE study were created in Microsoft Access by Jason Barber (barber@uw.edu). Databases for screening, outcome assessments and data validation are located on the Hemingway server here: T:\TBI Care\Databases.

Screening Database

The screening database is used for tracking potential participants from recruitment sources, storing screening and baseline data, randomizing participants and to create a consort report. A password is required for accessing the database on the server. Contact Jason Barber to obtain the password.

Tracking Attempts

All attempts and communications with participants should be logged in the "Attempts" tab of the screening database.

New Participant

Whenever a new potential participant is referred to the TBI-CARE study, or a potential participant is found during the clinic schedule review, they should be added to the Screening database to ensure they are screened for potential participation.

1. Open TBI-CARE Screening Database
2. Click on the button labeled "Add New Participant"
3. Enter as much information as is available. Fields include:
   a. *First Name*
   b. *Last Name*
   c. *HMC #/ UWMC #* (patient ID)

d. *Recruited via*: this is how the participant was referred. If the participant called in expressing interest, be sure to ask how they heard about the study (i.e. flyer in exam room at UW/HMC, PT Referral, etc.).
e. *Letter Date:* Indicate the date a recruitment letter was sent out for those recruited via clinic schedule review.
f. *Deferral Date:* List the date for the end of deferral period (used for potential participants who have are not 6 months post injury or who have otherwise indicated they would like to be contacted at a later date).
g. *Date of birth*
h. *Date of injury*
i. *Sex*
j. *Cause of injury:* List specific details in the details comment box.
k. *Chart-Rev Eligibility:* Indicate if the participant is eligible based on the pre-screening chart review. For participants who are NOT eligible after chart review, indicate in the "Why Eligible" drop down box why the participant has been excluded.
l. *Why Eligible:* Indicate eligibility status by selecting the appropriate choice in the drop-down box.

4. The new participant will appear in the window on the left side of the database screen.

Status

A participant's status will be indicated in the far-right column of the participants list box. Participants may be displayed and sorted according to the categories listed above in the shaded gray boxes.

Letter

Once a participant has been pre-screened by chart review, a letter will be sent and the participant's status will appear as "Letter" in the screening database. Once an attempt is logged in the attempts field, the status will automatically change to "Pending."

Pending

For participants who are still pending follow-up, their statuses will appear as "Pending" in the database until further delineated by another sub-group.

Pending-Active(A)

Status indicates the research team is actively communicating with the participant—may denote that a screening interview has been scheduled.

Pending-Backburner(B)

Status indicates the research team is not actively making attempts to call this person, but the participant should be kept on the follow-up call lists. For example, if a clinician does not think a potential participant would be eligible for the study, that person may be put on the backburner as low priority for a screening.

Pending-Clinic Watch(C)

Status indicates no active phone calls need to be made for this participant, but a member of the research team will attempt to meet the participant during an upcoming clinic appointment.

Deferred

For participants who have not yet reached their 6-month injury anniversary, their statuses will appear as "Deferred" and the deferral date in the screening database will denote when the participant is eligible for recruitment. A deferral status may also be used to indicate a potential participant would like to be contacted at a future date. Once the deferral period is up, the database will automatically change the status to "Pending."

Inactive

For participants who have had at least three call attempts and one clinic visit attempt, but no contact has been successful, their status can be changed to "Inactive" in the database. No further action is required for "Inactive" participants.

Ineligible

For participants who have been pre-screened, screened or rescreened and found to be ineligible for the study. Participants may be changed from ineligible at a later time, if circumstances require (i.e. the research team decides to screen past participants again in the future).

Eligible

For participants who have completed the screening interview and are eligible to join the study. Once a participant has completed the screening, their status should be changed under the "Why Ineligible" drop down box to "Eligible." A participant's status will remain as "Eligible" until the signed consent forms are received.

Consented

For participants who have returned signed consent and HIPAA Authorization forms, their statuses should be changed to "Consented" in the screening database and the date of consent should be updated. A participant's status will remain "Consented" until the baseline is administered and randomization occurs.

Randomized

Once a participant has completed the baseline interview, the randomization process will occur and the participant's status will automatically change to "Randomized" in the database. The randomization sequence is in blocks of 4 and is embedded in the screening database such that once a subject is enrolled and entered into the database, the examiner will just click the "randomize" button and the CCM will be notified. No further action is needed for Randomized participants in the screening database. Outcome assessments are tracked in a separate database (see Outcome Tracking Database section for more information).

Data Entry

All screening interview data must be entered into the screening database (for both eligible and ineligible participants).

1. Select the participant you want to enter data for in the "participants displayed" box.

2. Click the first tab to begin entering screening data.
3. Data will automatically save when you click to each new tab.
4. Add comments to the “Screening comments” section. Include:
    a. Date of upcoming clinic appointment
    b. Time of appointment
    c. Name of provider
    d. Location of the visit (HMC, UWMC, or other clinic)
    e. Other notes or information gathered during the screening interview that may be helpful for the care manager.
5. *If ineligible, choose one of the dropdown choices in the in “Why Ineligible” menu.*

Consort Flow Report

A report showing the breakdown of participant status from referral to randomization can be generated by clicking on the “Consort Flow” button in the TBI-CARE Screening database. A new report should be generated for the monthly TBIMS meeting and saved in the folder T:\TBI Care\Reports
For assistance with the report, contact Jason Barber <barber@uw.edu>.

Outcome Tracking Database

The Outcome Database is used to track follow-up assessments due. Participants will appear in the pending records displayed as soon as their follow-up interview windows open. The tracking database is also used to link to participant contact info, and for data entry.

No notifications are sent to alert study staff when assessment windows open. The database must be checked regularly to check for assessments due.

Baseline Assessment

Baseline Assessments will be conducted over the telephone after a signed consent form and HIPAA form have been received by study staff. The baseline may also be administered during a clinic visit if it is more convenient for the participant.

Baseline assessments must be administered within two weeks of passing the screener. If more than 2 weeks have elapsed, they must be asked to rate their pain on average over the past week on a 0-10 scale. If their score is 4 or greater, proceed with administering the baseline. If their score is less than 4, they are ineligible and baseline should not be administered.

Once the baseline assessment has been completed, randomization to either the Collaborative Care (CC) or Usual Care (UC) group will take place no greater than 2 weeks from date of baseline. Participants will be contacted by the Care Manager in order to inform them as to which group they have been assigned. Those participants assigned to the CC group will schedule their first telephone call (or in-person meeting) with the Care Manager (CM) (Mary Curran). Those in the U C group will be provided with information about addressing their desired area(s) of concern with their healthcare provider by the CM.

Upon completion of the baseline assessment, the examiner must stress the importance of remaining blinded as to which group (intervention vs. usual care) the participant is randomized. If a member of the data collection team is unblinded by a participant, then an alternate team member should be utilized for future follow-up assessments.

Baseline Rescreening

To ensure that participants are still eligible at the time of the baseline interview, a **Rescreening form** will be administered at the beginning of the interview. If a participant is no longer eligible after the rescreening portion of the baseline interview, they will be informed of their ineligibility status and a baseline assessment will not be administered. Payment of $5 will be sent, along with any additional resources that may be helpful to the participant (i.e., flyer for other research studies, POP Pain Study).

Data Entry

All baseline interview data must be entered into the screening database before randomization can occur.

1. Select the participant you want to enter data for in the "participants displayed" box.
2. Click the "Baseline Assessment" button to begin entering data.
3. Data will automatically save when you click to each new tab.
4. Add comments to the "General Comments and Item Details" section on page 10 of the data entry screen.
5. Check the box indicating baseline should be considered "completed." **This box was added in the event some baseline questions are skipped or omitted by the participant.* ***The participant cannot be randomized until this box has been checked.***

Randomizing a Participant

Participants are randomized after the baseline assessment is completed, and it is determined that they are eligible for enrollment.

1. Open the TBI-CARE Screening database.
2. Enter the Baseline Assessment by clicking the "Baseline Assessment" button at the top of the database. ****Check the completed box before closing the data entry window.***
3. Select the Randomization status.
    a. If the participant meets criteria for randomization select "0-OK to Randomize."
    b. If the participant is ineligible for randomization, select "1-Do not Randomize."
4. For ineligible participants, stop here.
5. Click on the "Get Next ID" button to get a new ID number.
6. Click on the "Randomize!" button that appears.
7. The participant's status should change to "Randomized."
8. Create a participant file with the Randomized participant number as the Participant ID. Files are stored in the second drawer of the locker cabinet closest to the printer.
9. Send an email to Care Manager, Mary Curran <mcurran@uw.edu> and cc Leslie Kempthorne <ette@uw.edu> informing her that a new participant has been randomized (see below for required information).

Create a Physical File

After randomization has occurred in the database, a physical file for the participant should be created. File folders are stored in the second drawer of the locker cabinet closest to the printer. Empty file folders are in the back, along with labels for creating the next participant file. Screening interviews should be placed on the left side of the folder and the baseline on the right

side. All 4- and 8-month follow-up assessments should be placed on the right side (most current forms on top).

Notifying Care Manager of Randomization

1. Create a new email message to Care Manager, Mary Curran (cc Leslie Kempthorne).
2. The participant line should be something like “TBI-CARE Participant Randomized.”
3. Include the Screening ID# of the randomized participant, as well as notes from the screening database regarding:
    a. Date of upcoming clinic appointments
    b. Time of appointment
    c. Name of provider
    d. Location of the visit (HMC, UWMC, or other clinic)
    e. Other notes or comments from the Screening or Baseline interview (including notes about ROI information)
    f. Indicate whether the participant would prefer to be notified by email (or standard mail) if randomized to the Usual Care group.
4. After all data has been entered into the screening database and forms are completely filled out (including CHART REVIEW section regarding injury level AND type on the screening form).
5. If the data is not entered in a timely fashion the Care Manager (Mary Curran) will request for review.
6. Mary will keep all files for a period of time before returning them, so as not to reveal which participants are in the intervention vs. Usual Care group.

Randomization Flow Chart

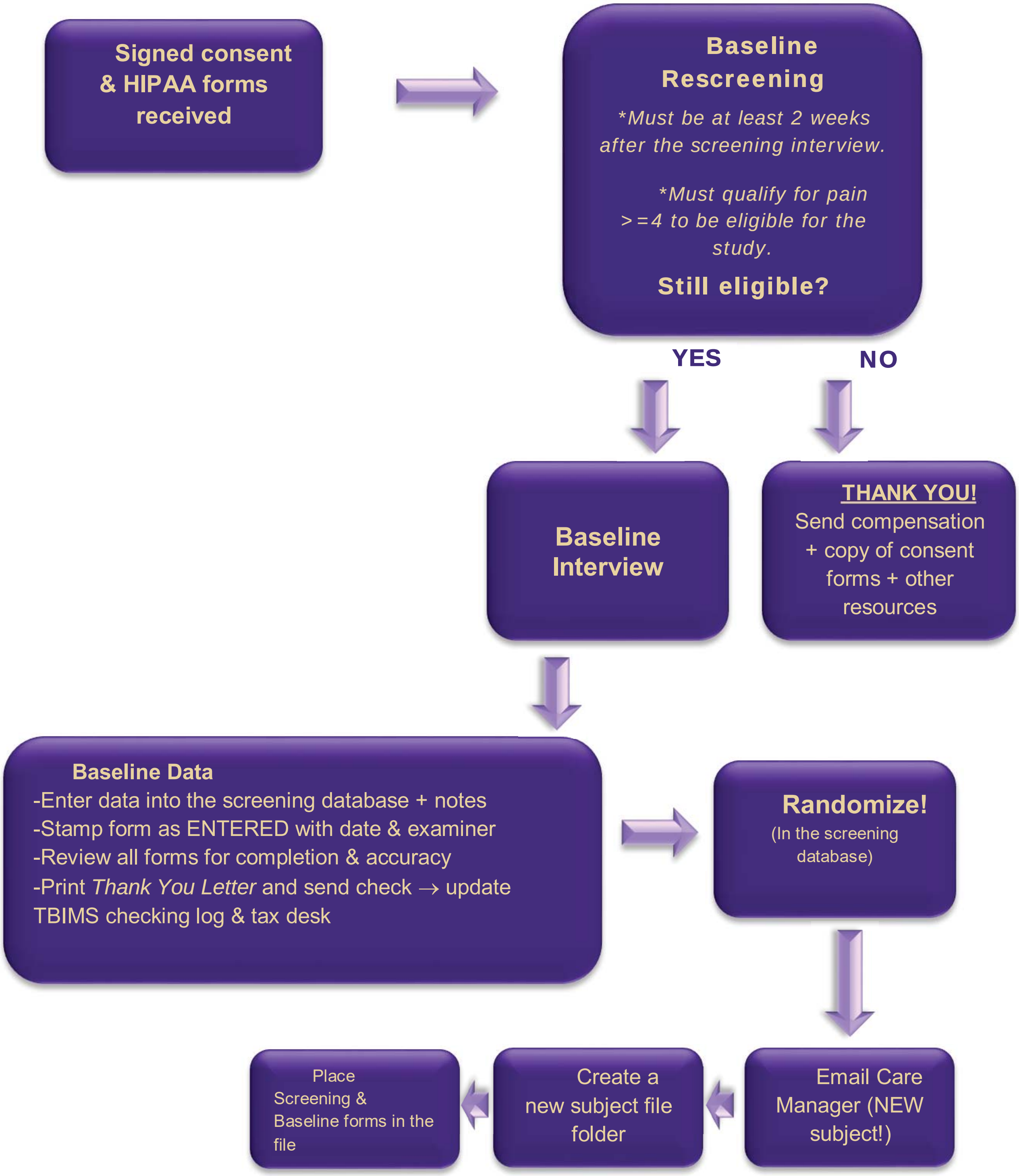

Outcome Assessments

All participants, regardless of group assignment, will be asked to complete an outcome assessment at 4 months and 8 months. The completion windows for the 4- and 8-month assessments are open for two weeks on either side of the actual date of assessment. Generally, staff should not begin calling participants for the assessment before the window opens but can keep calling after the window closes *(about 1 month grace period after the window closes but confirm with PI on case-by-case basis)*.

Adding Participant Contact Info

1. Once a participant has been randomized, they appear in the outcome tracking database as due for the 4- or 8-month assessment.
2. Select the participant in the “Records Displayed” window.
3. Click on the “Contact Info” button.
4. If the participant does not appear in the window, you may need to select the appropriate “Display” or “Include” types from the options above.
5. Enter the participant’s contact information.
6. The information will be automatically saved upon closing the contact info window.

Data Entry

1. Select the participant and the month you want to enter data for in the outcome database.
2. Click the “data entry” button.
3. The ID number and month that you are entering data for will appear at the top of the new window.
4. Click on the button for each measure to input data.
5. Data will automatically save when you click “close” out of each measure.

Quality Assurance

All forms entered for data collection will be reviewed by a staff member to ensure correct entry. If a discrepancy or error is found it will be flagged and brought to the staff who entered the data and or group for discussion.

TBI-CARE Study Staff

PI: Jeanne Hoffman, PhD	jeanneh@uw.edu	206-221-6511
Co-I: Sylvia Lucas, MD, PhD lucass@uw.edu		
Co-I: Jesse Fann, MD, MPH fann@uw.edu		
Co-I: Jennifer Zumsteg, MD zumsteg@uw.edu		
Consultants:		
Charles Bombardier, PhD	chb@uw.edu	
Dawn Ehde, PhD	ehde@uw.edu	
Collaborative Care Manager:		
Mary Curran	mccurran@uw.edu	206-685-1666
Research Coordinator:		
Laurie Peabody	lpeabody@uw.edu	206-744-3607
Research Manager:		
Leslie Kempthorne	ette@uw.edu	206-685-1082
Research Assistants:		
Meghan Gill, BA	mg63@uw.edu	
Silas James, MPA	silast@uw.edu	
Database Manager		
Jason Barber, MS	barber@uw.edu	
