# Supplementary material for: Collaborative Care for Chronic Pain After Traumatic Brain Injury: A Randomized Clinical Trial
Source: JAMA Netw Open. 2024 Jun 3;7(6):e2413459. doi: 10.1001/jamanetworkopen.2024.13459 (PMC11148690; doi:10.1001/jamanetworkopen.2024.13459)
Supplement: Supplement 2. — Data Sharing Statement [file jamanetwopen-e2413459-s002.pdf]

Data Sharing Statement

Hoffman. Collaborative Care for Chronic Pain After Traumatic Brain Injury. *JAMA Netw Open*. Published June 03, 2024. doi:10.1001/jamanetworkopen.2024.13459

Data
Data available: Yes
Data types: Deidentified participant data
How to access data: jeanneh@uw.edu
When available: With publication

Supporting Documents
Document types: None

Additional Information
Who can access the data: Researchers whose proposed use of the data has been approved.
Types of analyses: For a specified purpose.
Mechanisms of data availability: with signed data access agreement
